# Sustained Release of Dexamethasone from 3D-Printed Scaffolds Modulates Macrophage Activation and Enhances Osteogenic Differentiation

**DOI:** 10.1021/acsami.3c09774

**Published:** 2023-11-28

**Authors:** Majed Majrashi, Anna Kotowska, David Scurr, Jacqueline M. Hicks, Amir Ghaemmaghami, Jing Yang

**Affiliations:** † School of Pharmacy, University of Nottingham, Nottingham NG7 2RD, U.K.; ‡ School of Life Sciences, University of Nottingham, Nottingham NG7 2RD, U.K.; § Nanoscale and Microscale Research Centre, University of Nottingham, Nottingham NG7 2RD, U.K.; ∥ Biodiscovery Institute, University of Nottingham, Nottingham NG7 2RD, U.K.

**Keywords:** tissue engineering, controlled-release, dexamethasone, 3D printing, macrophages, mesenchymal stem cells, immunomodulation

## Abstract

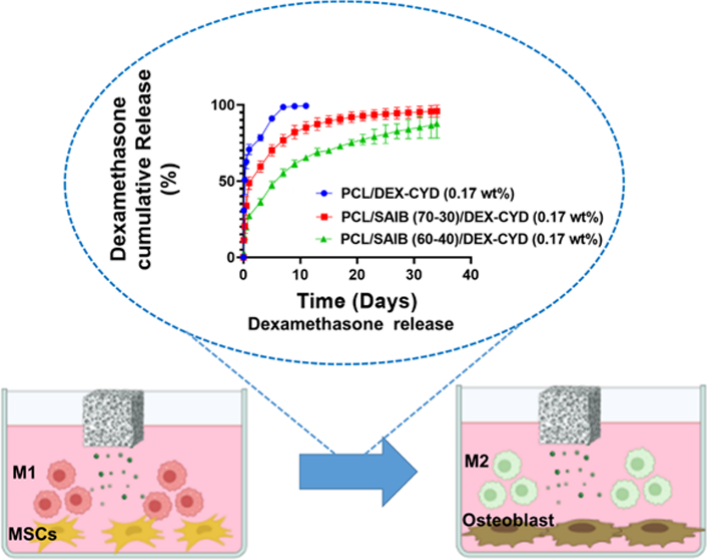

Enhancing osteogenesis via modulating immune cells is emerging as a new approach to address the current challenges in repairing bone defects and fractures. However, much remains unknown about the crosstalk between immune cells and osteolineage cells during bone formation. Moreover, biomaterial scaffold-based approaches to effectively modulate this crosstalk to favor bone healing are also lacking. This study is the first to investigate the interactions between macrophages and mesenchymal stem cells (MSCs) in co-cultures with the sustained release of an anti-inflammatory and pro-osteogenesis drug (dexamethasone) from three-dimensional (3D)-printed scaffolds. We successfully achieved the sustained release of dexamethasone from polycaprolactone (PCL) by adding the excipient-sucrose acetate isobutyrate (SAIB). Dexamethasone was released over 35 days in the 17–163 nM range. The osteogenic differentiation of MSCs was enhanced by M1 macrophages at early time points. The late-stage mineralization was dominated by dexamethasone, with little contribution from the macrophages. Besides confirming BMP-2 whose secretion was promoted by both dexamethasone and M1 macrophages as a soluble mediator for enhanced osteogenesis, IL-6 was found to be a possible new soluble factor that mediated osteogenesis in macrophage-MSC co-cultures. The phenotype switching from M1 to M2 was drastically enhanced by the scaffold-released dexamethasone but only marginally by the co-cultured MSCs. Our results offer new insight into macrophage-MSC crosstalk and demonstrate the potential of using drug-release scaffolds to both modulate inflammation and enhance bone regeneration.

## 1 Introduction

Bone tissue engineering commonly utilizes biomaterial scaffolds that can accommodate stem cells and/or molecules that promote angiogenesis, stem cell recruitment, and osteogenic differentiation.^1,2^ The responses of bone-forming stem/progenitor cells to biomaterial scaffolds have been the focus of this field. Increasingly, the interactions between osteolineage cells and immune cells and the inflammatory responses associated with implanted scaffolds are attracting more attention.^3,4^ It is now well known that immune cells play critical roles in bone healing and homeostasis.^5^ The early inflammation is essential for removing debris and dead cells, for promoting angiogenesis, and for recruiting cells to the fracture site.^6^ However, this inflammation needs to subside and resolve to allow for proper bone formation. Persistent inflammation without returning to the normal homeostasis status is associated with impaired tissue repair and fibrosis. The presence of a biomaterial scaffold increases the complexity of the immune microenvironment at a bone defect site due to immune cell–biomaterial interactions.^7^ On the other hand, biomaterials offer an opportunity to modulate local immune responses toward a favorable condition for tissue regeneration.^8,9^ Bone healing depends on coordinated interactions between immune cells and osteogenic stem/progenitor cells.^3^ Macrophages have been demonstrated to be critical players during fracture repair. Animal studies have shown that fractures do not heal without the direct involvement of macrophages.^10^ Efficient fracture healing relies on the migration of bone marrow-derived monocytes/macrophages to the fracture site.^11^ Bone resident osteomacs have also been demonstrated as critical mediators of endochondral and intramembranous bone healing.^12,13^ Co-cultures of mesenchymal stem cells (MSCs) with macrophages have shown that M0 and M1 macrophages solely stimulated the osteogenic differentiation of MSCs in the early and middle stages (measured by ALP activity and gene expression of early-stage osteogenic markers) while M2 induced higher late-stage mineralization (4 weeks in vitro).^14^ However, in another study, both M1 and M2 macrophages were found to promote similar levels of bone mineralization in MSCs after 4 weeks of co-culture.^15,16^ Subjecting MSCs to a 72 to 96 h pro-inflammatory environment (M1) before polarizing macrophages to M2 by using IL-4 was found to be critical for optimal mineralization in vitro.^17^ Bone marrow-derived macrophages encapsulated in a hydrogel were found to promote the osteogenic differentiation of bone mesenchymal stem cells.^18^ Oncostatin M (OSM) has been identified as a soluble factor that is secreted from M1 macrophages via a COX-2 and PGE2 regulatory loop for promoting osteogenic differentiation of MSCs.^5^ Taken together, the literature generally shows that the initial inflammation and presence of M1 macrophages and subsequent macrophage transition to M2 are important for osteogenic differentiation and mineralization. Conflicting data in the literature also necessitate more investigation on this topic.

However, these studies have only investigated the effects of macrophages on MSCs in vitro by adding cytokines directly to culture media to modulate macrophage polarization. In the bone tissue engineering context, this switch of macrophage polarization using soluble factors would need to be achieved in situ. To this end, incorporation and sequential release of M1 and M2-promoting molecules (IFN-γ, IL4) from decellularised bovine bone scaffolds was demonstrated to promote initial M1 polarization and subsequent transition to M2, which enhanced vascularization within the scaffolds.^19^ A similar approach for titania nanotubes showed increased M1 and M2 cytokines at early (3 days) and late time points (7 days), respectively, in vitro.^20^ Black phosphorus incorporated in PLGA recruited and stimulated M2 macrophages and promoted hBMSCs proliferation and differentiation.^21^ PEG-based hydrogels loaded with IL10 and aspirin-triggered resolvin-D1 (both for inflammation resolution) have been shown to polarize macrophages toward an anti-inflammatory M2 phenotype.^22^ Anti-inflammatory silibinin has been encapsulated in GelMA to promote M2 macrophage polarization.^23^ It is worth noting that these studies all showed a relatively quick release of inflammation-modulating soluble factors. Anti-inflammatory drugs, such as dexamethasone, have been loaded in three-dimensional (3D) printed or electrospun polymer scaffolds to modulate inflammation or promote osteogenic differentiation.^24,25^ Despite its known adverse effect on bone remodeling at supraphysiological concentrations,^26^ 10–100 nM dexamethasone has been widely used to promote osteogenic differentiation of MSCs in vitro.^27^ The release profile of loaded dexamethasone is polymer matrix dependent, with PLA delivering a relatively more sustained release compared to burst release from polycaprolactone (PCL).^25^ Moreover, the burst release of dexamethasone from PCL did not reduce inflammatory capsule compared to drug-free PCL electrospun fibers,^24,25^ suggesting a sustained release of dexamethasone is necessary for modulating inflammation.

To the best of our knowledge, the interplay between macrophages and MSCs under controlled-release scaffolds has not been investigated. Given the potential of immunomodulatory biomaterial scaffolds in orthopedics and other fields, studies like the one presented here will offer new insight into this important topic of macrophage-MSC crosstalk. Herein, we studied the responses of macrophages and MSCs in co-cultures with the presence of controlled-release PCL scaffolds to mimic the case of bone healing with the presence of an immunomodulatory biomaterial scaffold. We hypothesized that the localized release of low-concentration (nM range) dexamethasone from scaffolds not only promotes osteogenic differentiation but also modulates macrophage activation status to favor bone formation. A suite of techniques was used to characterize the drug distribution and release profiles. The polarization of macrophages and osteogenic differentiation of MSCs were investigated in macrophage-MSC co-cultures.

## 2 Materials and Methods

### 2.1 Materials

Polycaprolactone (PCL, Mn 80,000), fetal bovine serum (FBS), mercaptoethanol, penicillin/ streptomycin, 70% ethanol, T75 tissue culture flask, 24-well tissue culture plates, papain, dexamethasone-cyclodextrin DEX-CYD, mass ratio 65:1000 = dex:(dex + cyd), granulocyte-macrophage colony-stimulating factor (GM-CSF), lipopolysaccharide (LPS) from *Escherichia coli*, macrophage colony-stimulating factor (M-CSF), Interleukin 4 (IL-4), methanol, dichloromethane (DCM), cetylpyridinium chloride (CPC), Alizarin Red, nonessential amino acids, β-glycerophosphate, and ethylenediaminetetraacetic acid (EDTA) were purchased from Sigma-Aldrich, U.K. THP-1 cell line (ATCC no. TIB-202), phorbol 12-myristate-13-acetate (PMA), Quanti-iTTM Picogreen kit, mouse anti-human calprotectin antibody, Rhodamine red goat anti-mouse IgG (H + L) secondary Ab, Alexa Flour 488 goat anti-rabbit IgG (H + L) secondary Ab, and DAPI and RPMI-1640 were purchased from Fisher Scientific, U.K. α-MEM (BE12–169F), and ToxiLight nondestructive cytotoxicity bioassay kit were purchased from Lonza, U.K., and rabbit anti-human MR Ab from Abcam-U.K.

### 2.2 Ink Preparation and 3D Printing of Scaffolds

To prepare PCL: Dexamethasone-Cyclodextrin (DEX-CYD) scaffolds, DEX-CYD complex was added to DCM at three different concentrations (0.77, 1.54, 3.85 wt/wt % corresponding to 0.05, 0.1, and 0.25 wt/wt % of dexamethasone). PCL was then added to the drug solutions at a concentration of 57 wt %/vol (PCL/DCM). To improve the release profile of dexamethasone, excipients including poloxamers (F127, F68, and L31), Span80, and Sucrose acetate isobutyrate (SAIB) were added to PCL at two mass ratios (70:30 and 60:40, PCL:excipient). DEX-CYD was added at a concentration of 2.63 wt/wt % (equivalent to 0.17 wt %/wt dexamethasone). All inks were left on the roller overnight to completely dissolve prior to printing. Lattice scaffolds with a dimension of 10 × 10 × 5 mm^3^ were 3D-printed in the air (regenHU, Switzerland) through a tapered tip (160 μm internal diameter) with the following parameters: strut spacing of 0.52 mm, speed rate of 8 mm/s, 0.12 mm layer thickness, and 6 bar of pressure. Ultraviolet (UV) (245 nm) was used to sterilize the printed scaffolds for 20 min on each side. Our previous data showed that the DCM evaporated to very low levels (comparable to virgin PCL) in air and showed no negative effect on cells.^2^

### 2.3 Characterization of Printed Scaffolds

The topographies, diameters of printed struts, and pore size of the 3D-printed scaffolds were visualized by scanning electron microscope (JEOL JSM-6490LV, U.K.). Glass transition temperatures of various scaffold materials were obtained by using differential scanning calorimetry (DSC) (TA discovery Q 2000). Samples were weighed within the 5–10 mg range, loaded into Tzero Hermetic pans, and sealed with lids. The samples were then heated from 30 to 200 °C at 5 °C /min, with a flow rate of nitrogen gas of 50 mL/min.

### 2.4 Time-of-Flight Secondary Ion Mass Spectroscopy (ToF-SIMS)

Analysis was conducted by using a ToF-SIMS IV instrument (IONTOF, GmbH). For surface analysis, a Bi_3_^+^ beam energy of 25 keV and a pulsed target current of ∼0.3 pA were employed. Dual-beam dynamic SIMS was conducted using a 20 keV Ar_1900_ gas cluster ion beam (GCIB) with a target current of 10 nA as a sputter beam and 25 keV Bi_3_^+^ analysis beam with a pulsed target current of ∼0.3 pA. Noninterlaced depth profiling was employed, whereby the sputter and analysis ion beams operate at alternating intervals with 5 frames of sputtering per 1 analysis frame and a 0.5 s pause between the sputtering and analysis. A region of 500 μm × 500 μm was sputtered, and a 200 μm × 200 μm region was analyzed with pixel density 128 × 128.

### 2.5 In Vitro Release of Dexamethasone

All 3D-printed PCL scaffolds (10 mm x 10 mm x 5 mm) loaded with DEX-CYD were incubated in 10 mL of phosphate-buffered saline (PBS) (pH 7.4) at 37 °C. At each time point, the 10 mL of medium was completely withdrawn and replaced with 10 mL of fresh PBS. Dexamethasone concentration was measured by measuring the absorption at 241 nm using a UV–visible (UV–vis) spectrophotometer (Agilent Technologies, U.K.). The unknown concentrations were quantified based on the calibration curve of known concentrations of dexamethasone. Limit of detection (LOD) and limit of quantification (LOQ) were determined based on the blank (PBS) signal.
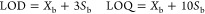
1where *X*_b_ is the mean and *S*_b_ is the standard deviation of the blank.

### 2.6 In Vitro Culture of MSCs and Macrophages

Bone marrow-derived human mesenchymal stem cells (MSCs) were immortalized, clonally selected, and maintained according to previous protocols.^28^ The MSCs have been regularly assessed to ensure the capability of expansion without a loss of trilineage differentiation potential. MSCs were incubated in an expansion medium Dulbecco’s modified Eagle’s medium (DMEM, high glucose) supplemented with 10% heat-inactivated fatal bovine serum (FBS), 1% nonessential amino acids, 1% l-glutamine, and 1% penicillin/streptomycin solution.

THP-1 monocytes were expanded in THP-1 cell culture medium (RPMI-1640 containing 10% heat-inactivated fetal bovine serum (FBS), 1% penicillin/streptomycin, 10 mM 4-(2-hydroxyethyl)-1 piperazineethanesulfonic acid (HEPES), 1 mM sodium pyruvate, 1% GlutaMax, 2.5 g/L glucose, and 0.05 mM mercaptoethanol). Monocytes were differentiated into M0 macrophages by 50 ng/mL of phorbol-12-myristate-13-acetate (PMA) for 6 h. After 6 h, the PMA medium was decanted and substituted with fresh complete growth media. Macrophages were rested in culture overnight at 37 °C and 5% CO_2_. For M1 polarization, M0 cells were exposed to 50 ng/mL Granulocyte-macrophage colony-stimulating factor (GM-CSF) and 100 ng/mL Lipopolysaccharide (LPS) and incubated for 72 h. For M2 polarization, M0 cells were exposed to 50 ng/mL macrophage colony-stimulating factor (M-SCF) and 20 ng/mL Interleukin-4 (IL-4) and incubated for 72 h.^29^

To evaluate the stability of macrophage phenotype in co-culture media composed of (50:50) (α-MEM: RPMI-1640) containing 10% FBS, 1% penicillin/streptomycin, and 1% Glutamax, THP-1 cells (5 × 10^5^ cells/well) were seeded into 24-well plates and differentiated to M0 M0 macrophages with PMA. Then, the cells were polarized into M1 and M2 for 3 days. At the end of day 3, polarization media were removed, and replaced with fresh media without polarization factors, and incubated for 7 days. At the end of each time point, supernatants were collected, and cells were fixed with 4% paraformaldehyde for immunostaining.

For macrophage-MSC coculturing on two-dimensional (2D) plastic wells, polarized M1 macrophages and MSCs were plated concurrently in a 5:1 ratio (2 × 10^5^ macrophages: 4 × 10^4^ MSCs) in a 6-well plate with 10 mL of medium. The co-culture used a mixed culture medium which was composed of 50% α-MEM and 50% RPMI-1640 and supplemented with 10% FBS, 1% antibiotic-antimycotic, 1% Glutamax, and 10 mM β-glycerophosphate. After the plating of macrophages and MSCs, 3D-printed scaffolds were added to and immersed in the cultures. Cell viability of macrophages and MSCs was determined by using the ToxiLight cytotoxicity assay (Lonza) according to the manufacturer’s instructions.

For macrophage-MSC coculturing in scaffolds, polarized M1 macrophages and MSCs were seeded concurrently in a 5:1 ratio (5 × 10^5^ macrophages: 1 × 10^5^ MSCs, suspended in 100 μL medium) onto each scaffold as we did in a previous paper.^2^ The seeded scaffolds were transferred to a new well plate to remove cells that were not maintained in the scaffolds. The cell-seeded scaffolds were cultured for 1, 7, and 14 days. At each time point, the supernatant was collected for ELISA. Cells on scaffolds were immunostained for calprotectin (M1), mannose receptor (M2), and DAPI (nucleus). The stained cells on scaffolds were imaged using a Zeiss CLSM 900 confocal microscope.

### 2.7 ALP Activity

Pierce *p*-nitrophenyl phosphate (PNPP) was used to evaluate the ALP activity according to the manufacturer’s recommendations. Cells were washed twice with 1× assay buffer. The cells were then lysed in 1 mL of 1% Triton-X-100 diluted in 1× assay buffer. 50 μL of supernatant was transferred to a 96-well plate. A 50 μL aliquot of PNPP was added to the supernatant and allowed to react for 30 min at room temperature. The absorbance was measured at 405 nm. Total ALP activity was calculated based on an ALP standard curve of known concentrations.

### 2.8 Alizarin Red Staining (ARS)

Alizarin red staining was used to measure mineralization. Cells were washed 3 times for 15 min at room temperature with PBS and fixed with 4% formaldehyde. Then, the cells were washed with deionized water and stained with 1 mL of 40 mM ARS per well for 30 min. Deionized water was used to wash the cells prior to imaging and quantification. The images of stained samples were collected with a Nikon SMZ1500 and a Nikon Digital sight DS-Fi2 camera (Nikon, Japan). Subsequently, stained cultures were quantified by destaining for 15 min at room temperature using 10% (w/v) CPC in 10 mM sodium phosphate at pH 7.0. The ARS concentration was measured by using the absorbance at 562 nm.

### 2.9 RUNX2 Gene Expression

RNA was extracted from cells using an RNeasy Plus Minikit (Qiagen) according to the manufacturer’s instructions. cDNA was synthesized from 1 μg of total RNA using a qPCRBIO cDNA synthesis kit (PCR Biosystems) according to the manufacturer’s instructions. qRT-PCR was performed on a QuantStudio 3 Real-Time PCR System. The primers used for qRT-PCR are listed in Table 1. Data were analyzed using Design and Analysis Software v2.6.0, QuantStudio 3. Relative expression of genes of interest was calculated by normalizing against the housekeeping gene glyceraldehyde 3-phosphate dehydrogenase (GAPDH) using the ΔΔCT Method.

**Table 1:** Primers Used in RT-PCR

genes	primers	sequence (5′ – 3′)
RUNX2	forward	GGAGTGGACGAGGCAAGAGTTT
	reverse	AGCTTCTGTCTGTGCCTTCTGG
GAPDH	forward	CTCTGCTCCTCCTGTTCGACA
	reverse	ACGACCAAATCCGTTGACTC

### 2.10 Cytokine Analysis

At each time point, incubation media were transferred to Eppendorf tubes and centrifuged at 13,000*g* and 4 °C to remove cellular debris. Supernatants were transferred to new Eppendorf tubes and frozen at −80 °C. TNF-α, IL-6, IL-10, TGF-β1, IL-1 β, and BMP-2 were quantified using ELISA according to the manufacturer’s guidelines (R&D Systems).

### 2.11 Immunofluorescent Staining

To evaluate M1 and M2 macrophage surface markers, cells were stained with 2 ug/mL mouse anti-human calprotectin antibody and 1 ug/mL rabbit anti-human mannose receptor antibody for 1 h, then stained with 8 μg/mL rhodamine red goat anti-mouse IgG (H + L) secondary antibody and 8 μg/ml Alexa Flour 488 goat anti-rabbit IgG (H + L) secondary antibody for 1 h. Cells were counterstained with 250 ng/mL DAPI. Cells were imaged with ZOE Fluorescent Cell Imager (Bio-Rad). Images were analyzed using ImageJ software (Fiji version).

### 2.12 Statistical Analysis

All values in this study were reported as mean or mean ± standard deviation (SD). Unless otherwise stated, statistically significant differences were analyzed using the one-way analysis of variance with Tukey’s post hoc test; *, **, ***, and **** indicate statistical differences with *p* < 0.05, *p* < 0.01, *p* < 0.001, and *p* < 0.0001, respectively. Detailed pairwise comparison tables are included in the Supporting Information.

## 3 Results

### 3.1 Morphology of 3D-Printed Scaffolds

**Figure 1:**
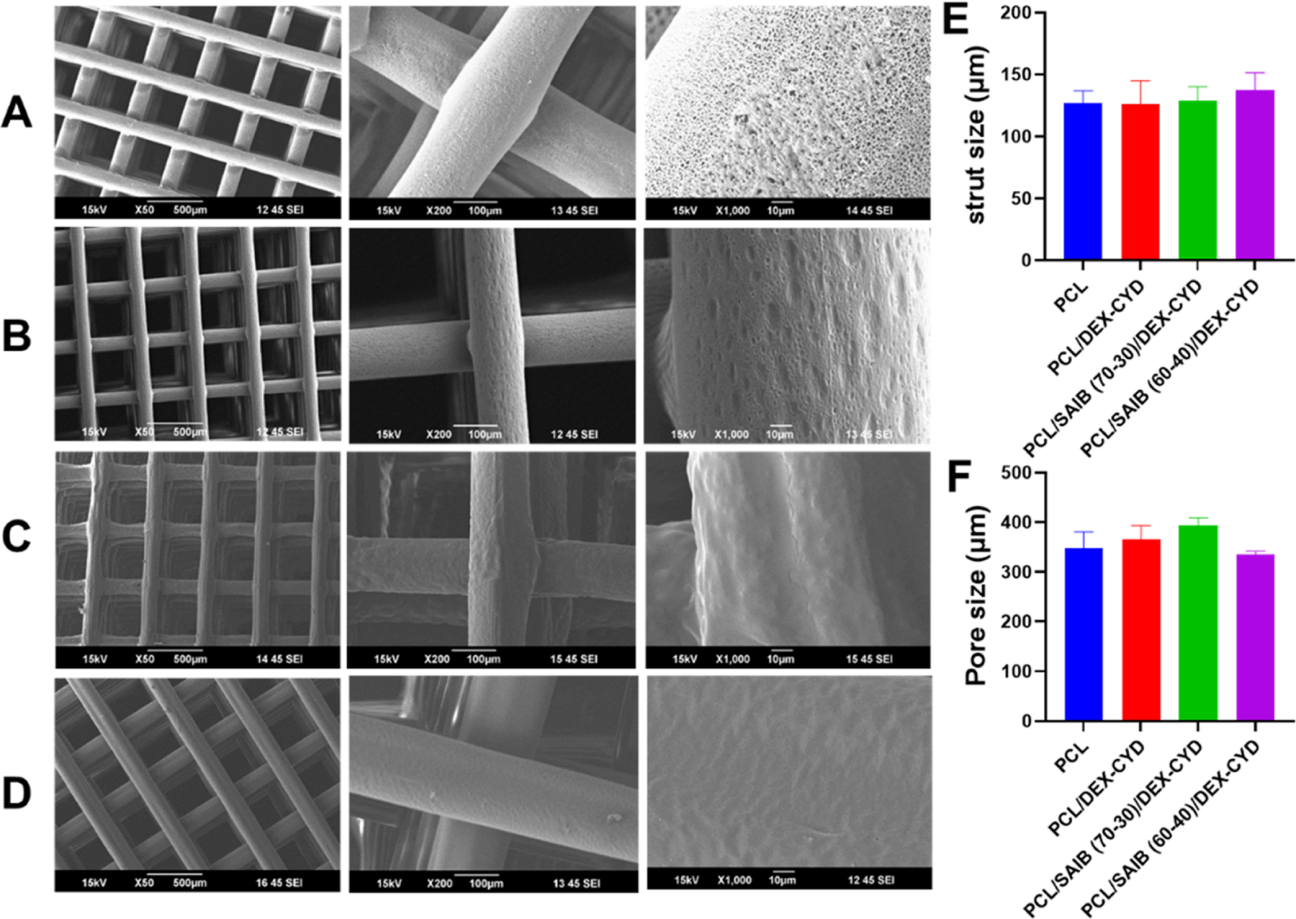
SEM images representing (A) PCL scaffold, (B) PCL/DEX-CYD, (C) PCL/SAIB (70:30)/DEX-CYD, and (D) PCL/SAIB (60:40)/DEX-CYD. (E) Strut diameter and (F) pore sizes (between struts). Results are presented as mean ± SD (*n* = 8).

The scaffolds were printed by the direct deposition of viscous PCL/SAIB/DEX-CYD solutions (a method sometimes referred to as direct ink writing^30^). The concentration of dexamethasone-cyclodextrin (2.53 wt %, equivalent to a dexamethasone concentration of 0.17 wt %) used in our study was the maximum amount that could be printed without clogging the nozzle. Figure 1A–D shows the morphologies of the printed scaffolds and the struts. Noticeably, PCL and PCL/DEX-CYD scaffolds showed pores on the strut surface. In contrast, pores were absent on the strut surface of the scaffolds containing SAIB. The pores were constrained only to the strut surface, as demonstrated in our previous study.^2^ These strut pores were possibly caused by solvent evaporation-induced phase separation, where competition between the phase separation dynamics and the solvent evaporation rate governs the microstructure formation.^31^ The addition of SAIB, which is an emulsifier, possibly perturbed the phase separation behavior, hence altering the microstructure on the strut surface. The addition of SAIB did not alter the strut size. The average strut diameter and pore size were similar across all scaffold groups (Figure 1E,F), which gave us confidence that varied drug release from these scaffolds was due to ink formulation, not geometry.

### 3.2 In Vitro Release of Dexamethasone from Scaffolds

**Figure 2:**
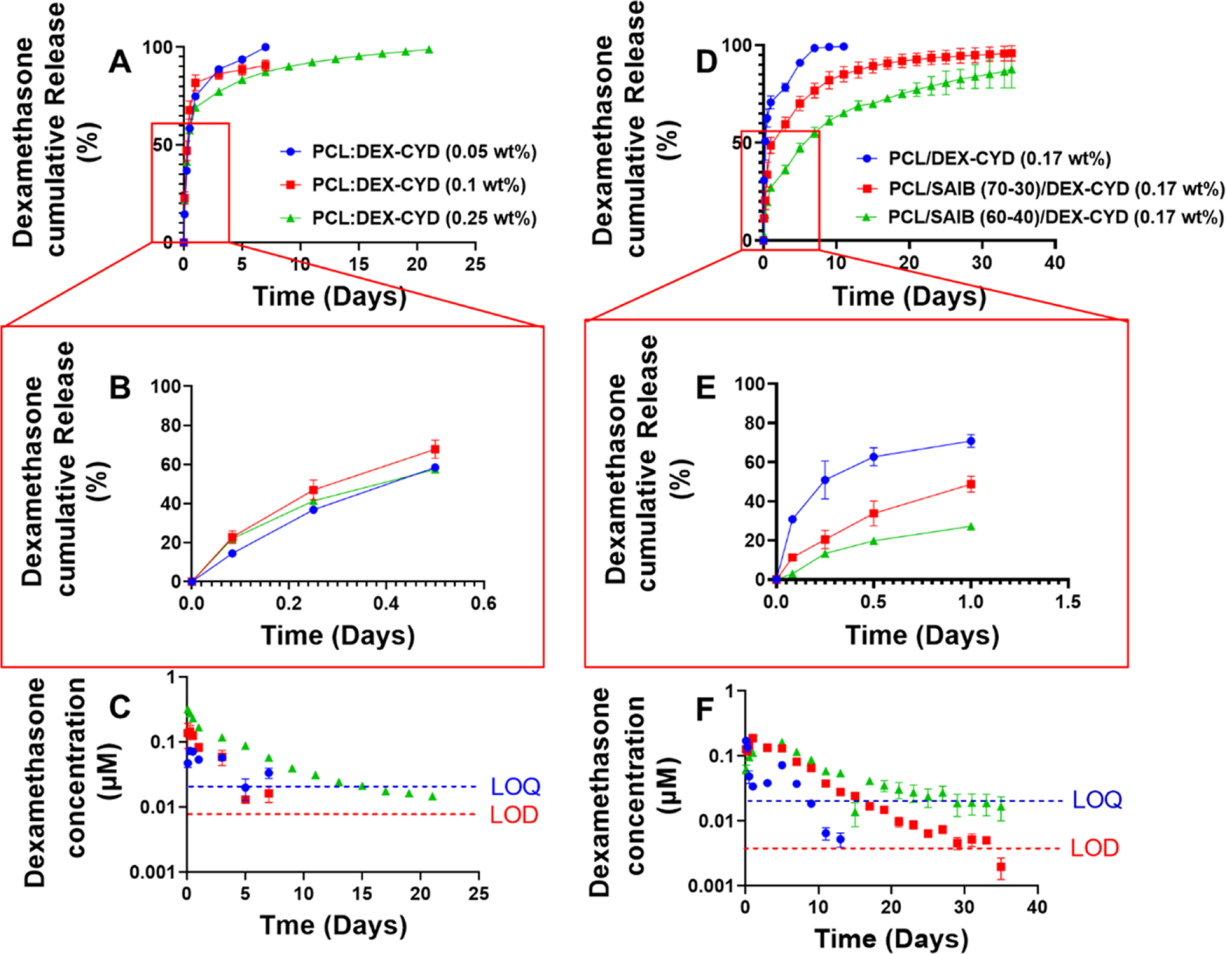
Cumulative and individual release at each collection time point in 10 mL of PBS. (A–C) 3D-printed PCL scaffolds loaded with 0.05, 0.1, and 0.25 wt % of dexamethasone. (D–F) 3D-printed scaffolds of PCL/DEX-CYD, PCL/ SAIB (70:30)/DEX-CYD, and PCL/SAIB (60:40)/DEX-CYD with 0.17 wt % dexamethasone. LOD, limit of detection. LOQ, limit of quantification. At each collection point, the whole release medium was replaced. Release medium was collected every 2 days after day 1. All data represent mean ± SD (*n* = 3).

The accumulative release and release at each collection time point of dexamethasone from all 3D-printed scaffolds are listed in Figure 2. All PCL/DEX-CYD scaffolds showed an initial burst release of over 50% in the first 12 h. The 0.05 and 0.1% dexamethasone scaffolds reached 100% cumulative release over 7 days. In contrast, scaffolds containing 0.25 wt % dexamethasone showed a longer release period and reached 100% cumulative release over 23 days. As shown in Figure 2C, the amount of drugs released at each individual collection time point was higher for the scaffolds with higher drug loading (0.25 wt %).

To reduce the burst release and prolong the release of dexamethasone, Pluronic F127, Pluronic F68, Pluronic L31, Span80, and SAIB, which all have surfactant properties, were added to PCL to test their ability to prolong drug release. Among the five tested excipients, SAIB showed the best ability to reduce burst release and prolong release time (Figure 2D–F and Figures S2–S4). SAIB was added to PCL with two different mass ratios (70:30, 60:40 PCL/SAIB) while keeping the same drug concentration (0.17 wt % dexamethasone). PCL/SAIB (70:30) and (60:40) showed approximately 30 and 19% burst release, respectively, in the first 12 h (Figure 2E), compared to 50% over the same time for PCL/DEX-CYD. The drug concentrations at each individual collection point are shown in Figure 2C,F. It is worth noting that some concentrations dropped below the limit of detection (LOD) or limit of quantification (LOQ) during the study. However, the released drug concentrations for the composition 60:40 (PCL/SAIB) were mostly above LOQ. The prolonged release of dexamethasone can potentially lead to improved effect as it maintains the necessary concentration long enough for modulating immune response and osteogenic differentiation.^24,32^

**Figure 3:**
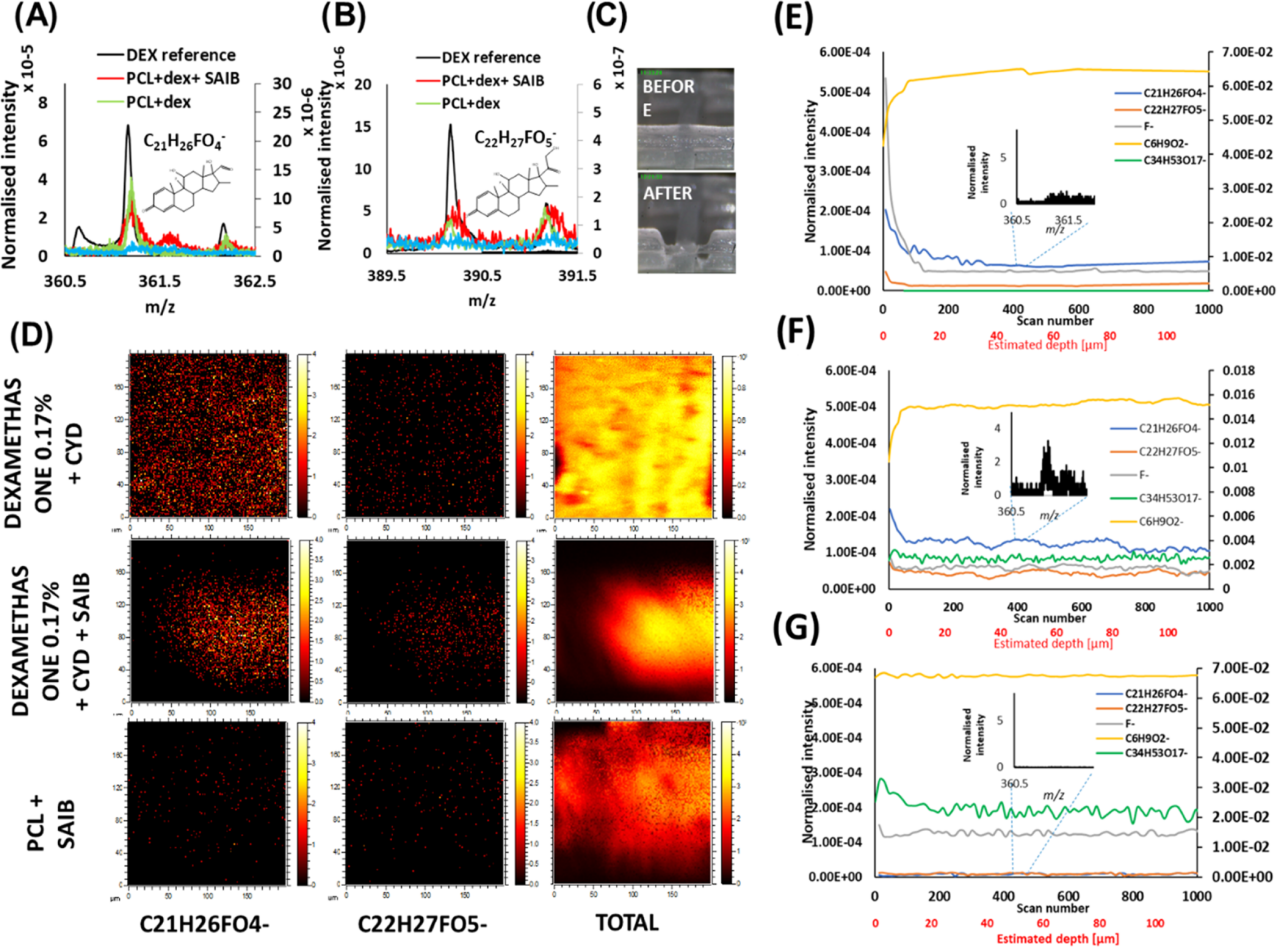
Dexamethasone distribution within polymer struts analyzed by using ToF-SIMS. (A, B) Spectra showing the two dexamethasone ion peaks. (C) Image of a strut before and after depth profiling. (D) Ion images of different individual struts. (**E–G**) Depth profiling of dexamethasone molecular ion C_22_H_27_FO_5_^–^ (orange) and fragment C_21_H_26_FO_4_^–^ (blue), F^–^ (gray), PCL marker C_6_H_9_O_2_^–^ (yellow), and SAIB marker C_34_H_53_O_17_^–^ (green) in three different scaffolds (E-PCL + dex; F-PCL + dex + SAIB; G-PCL + SAIB).

To explain the reason for improved release after adding SAIB, we analyzed the distribution of dexamethasone within different polymer matrices by using ToF-SIMS depth profiling. Two ions from dexamethasone ([M – H]^−^ C_22_H_27_FO_5_^–^ and fragment C_21_H_26_FO_4_^–^) were detected in the spectra of all dexamethasone-containing scaffolds but not in the PCL + SAIB reference scaffold as shown in Figure 3A,B. Dexamethasone was homogeneously distributed on the surfaces of the PCL + dex + SAIB scaffolds and the PCL + dex scaffolds (Figure 3D). Dexamethasone was demonstrated to be more concentrated on the strut surface of the PCL + dex scaffolds according to the depth profiling, which showed high dexamethasone ion intensity at the strut surface with a rapid signal intensity reduction into the inside of the strut (Figure 3E). The intensities of the two dexamethasone ions reduced to an almost undetectable level inside of the strut, evidenced by the flatness of the lines of the two dexamethasone ions (Figure 3E). In contrast, the intensity of the dexamethasone ions decreased slower from the surface to the inside of the PCL + dex + SAIB scaffolds (Figure 3F). The intensities of these dexamethasone ions were much higher inside the SAIB-containing scaffolds (nonflat signal with variations) than those in the PCL + dex scaffolds (Figure 3F).

Differential scanning calorimetry was also utilized to investigate the interaction between DEX-CYD, PCL, and excipients (Figure S1). The addition of DEX-CYD did not alter the melting point of PCL, while the addition of SAIB and other excipients decreased the melting point (Table S1), suggesting a plasticizer effect of these excipients by mixing with PCL.^33^ However, the calorimetric data could not show how homogeneous the drug and excipients were mixed within PCL. Depth profiling by ToF-SIMS was necessary to demonstrate the distribution of DEX-CYD and excipients in PCL.

### 3.4 Cell Viability and Proliferation in Different Media with and Without Scaffolds

**Figure 4:**
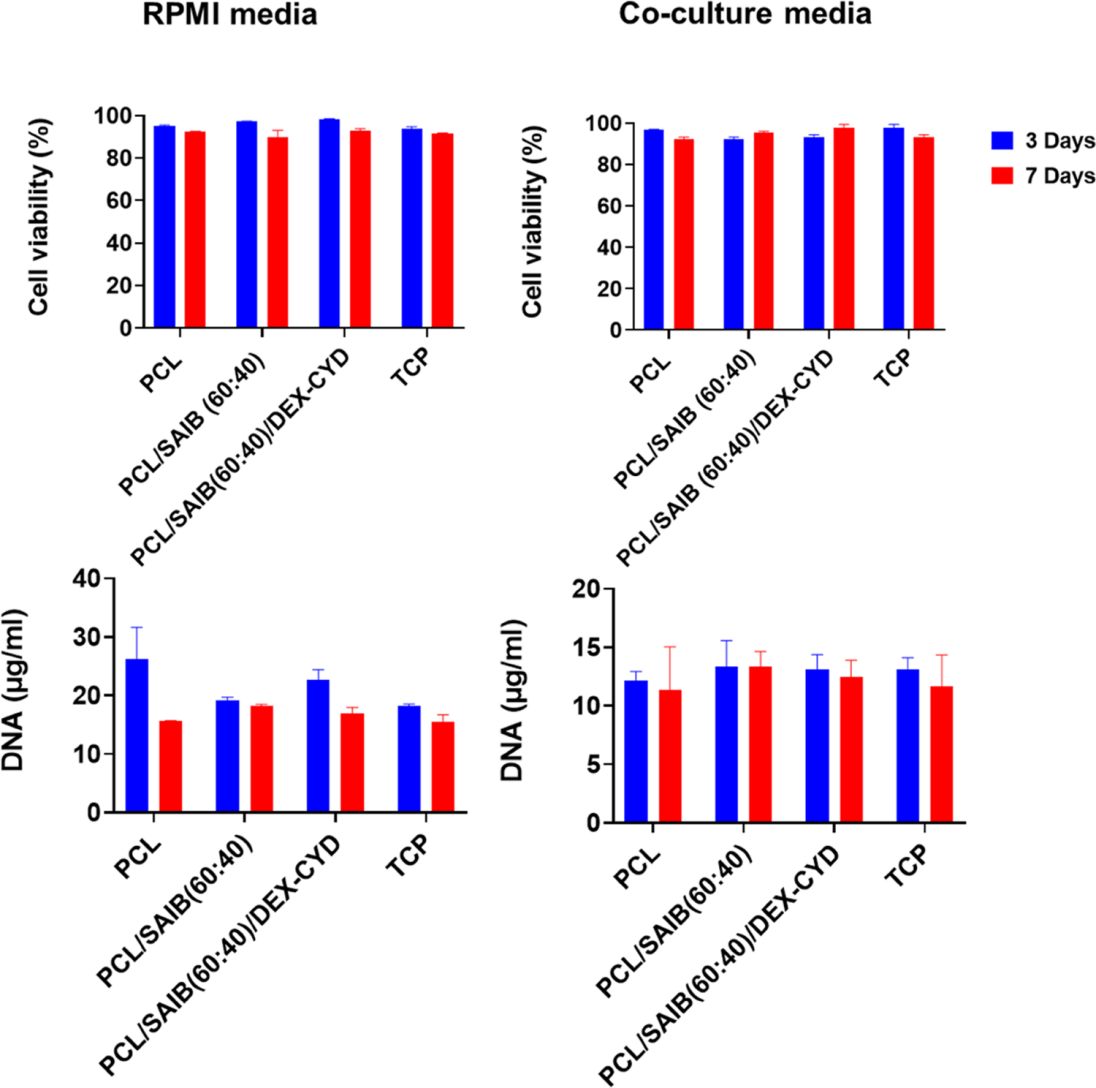
Cell viability and proliferation of THP-1-derived macrophages in RPMI and co-culture media (both media supplemented with LPS + GM-CSF) with and without scaffolds in the media. All data represent mean ± SD (*n* ≥ 3).

After achieving a more sustained release of dexamethasone, we first studied the cytotoxicity of this drug delivery system in macrophages and MSCs. Cell viability and proliferation were measured by the ToxiLight assay and PicoGreen assay, respectively. As co-culture will be carried out in a mixture of media (50:50 RPMI/α-MEM), the effect of the composition of culture media on cells was first tested. Cell viability (macrophages and MSCs) was similar between the single media and the co-culture media (50:50 RPMI/α-MEM) (Figure 4). In addition, there was no difference in the cell viability across all four sample groups for macrophages. Macrophages also did not proliferate, which was expected as these monocyte-derived macrophages do not proliferate after activation by PMA.^34^

**Figure 5:**
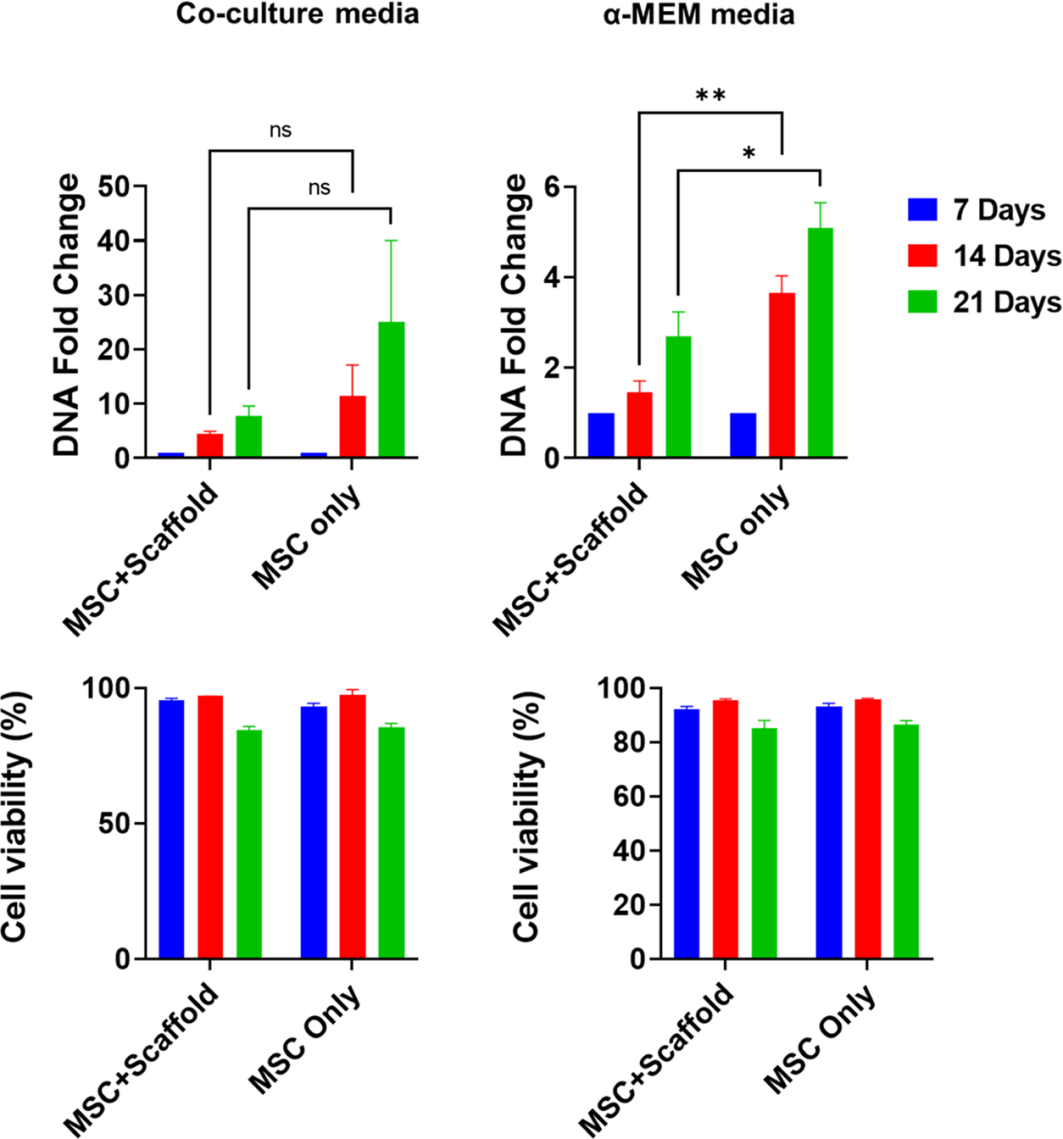
Viability and proliferation of MSCs in α-MEM and co-culture media with or without drug-loaded scaffolds. DNA fold change was normalized to day 7. All data represent mean ± SD (*n* ≥ 3). * *p* < 0.05, ** *p* < 0.01.

For the MSCs, the presence of scaffold (PCL + SAIB + dexamethasone) reduced cell proliferation compared to that of the MSC-only culture (Figure 5). This is likely caused by the released dexamethasone in the culture, which inhibited the proliferation of MSCs.^35^ The co-culture media enhanced the proliferation of MSCs compared to α-MEM media. This could be attributed to the broader range of nutrients in the co-culture media comparable to single media. The viability of MSCs was not affected by the drug-release scaffold or the medium composition. However, MSC viability decreased slightly at day 21 compared with days 7 and 14, which was likely due to cell confluency. We also confirmed that the co-culture media did not affect the differentiation of THP-1 monocytes to M0, M1, and M2 phenotypes compared to RPMI media (Figures S5 and S6).

### 3.5 Immunomodulation of Macrophages by Bolus and Scaffold-Released Dexamethasone

**Figure 6:**
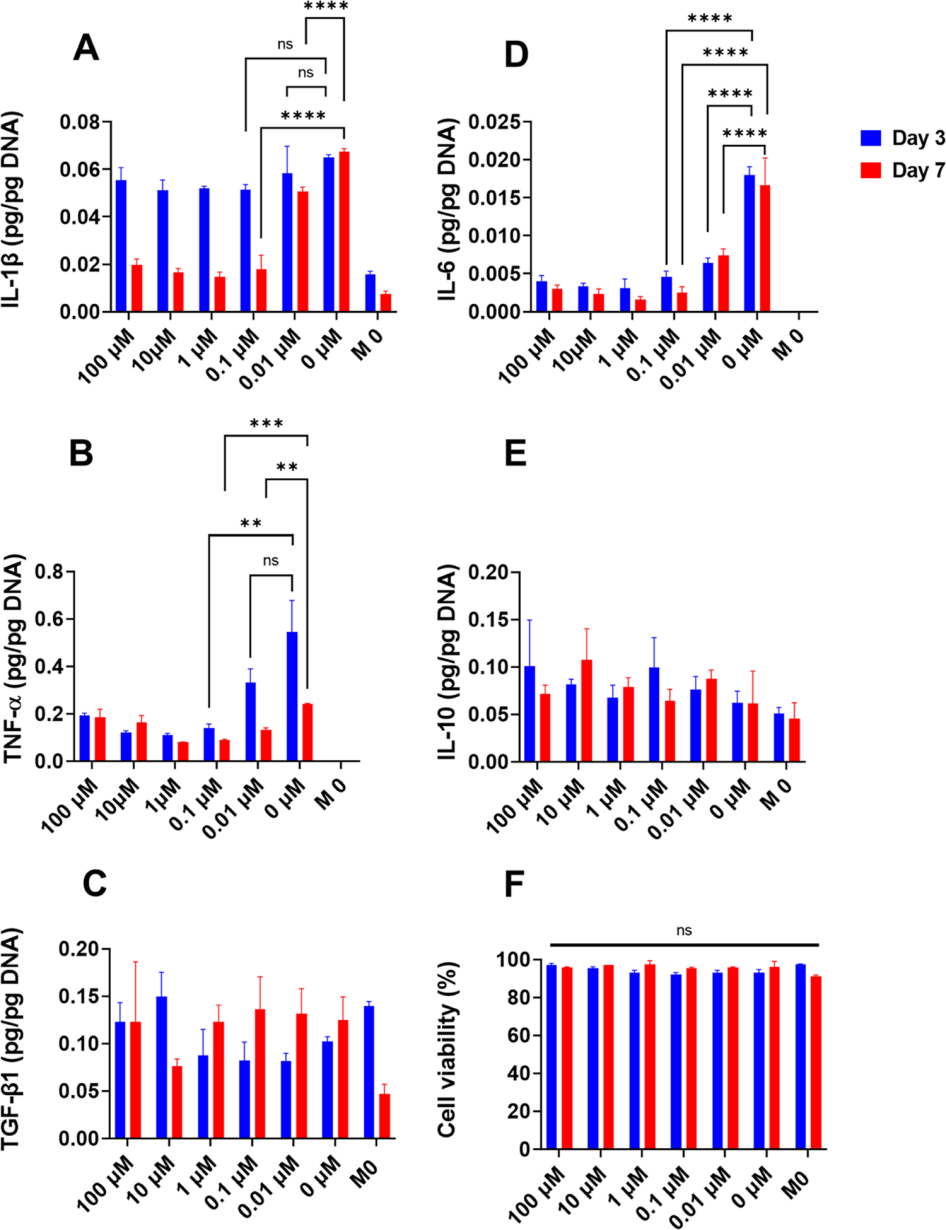
Effect of dexamethasone concentration on macrophage polarization and viability. THP-1 cells were first differentiated into M0 macrophages with PMA. LPS, GM-CSF, and dexamethasone were then added together to the media (except the M0 control). All data represent mean ± SD (*n* ≥ 3). * *p* < 0.05, ** *p* < 0.01, *** *p* < 0.001, and **** *p* < 0.0001.

Before studying the interactions between macrophages and MSCs in co-cultures, we characterized the polarization of macrophages by dexamethasone in single cultures. Different amounts of dexamethasone were directly added (bolus) to culture media to determine the effective concentrations that can modulate macrophage polarization. Figure 6 shows that the effective concentration of dexamethasone in suppressing M1 cytokines was approximately in the 10–100 nM range (depending on the specific M1 cytokine). The M2 markers (IL-10 and TGF-β1) were not noticeably affected by dexamethasone concentrations during the 7-day cultivation. This suggested that the suppressive effect of dexamethasone on M1 phenotype was relatively quick compared to its effect on switching macrophages to the M2 phenotype.

**Figure 7:**
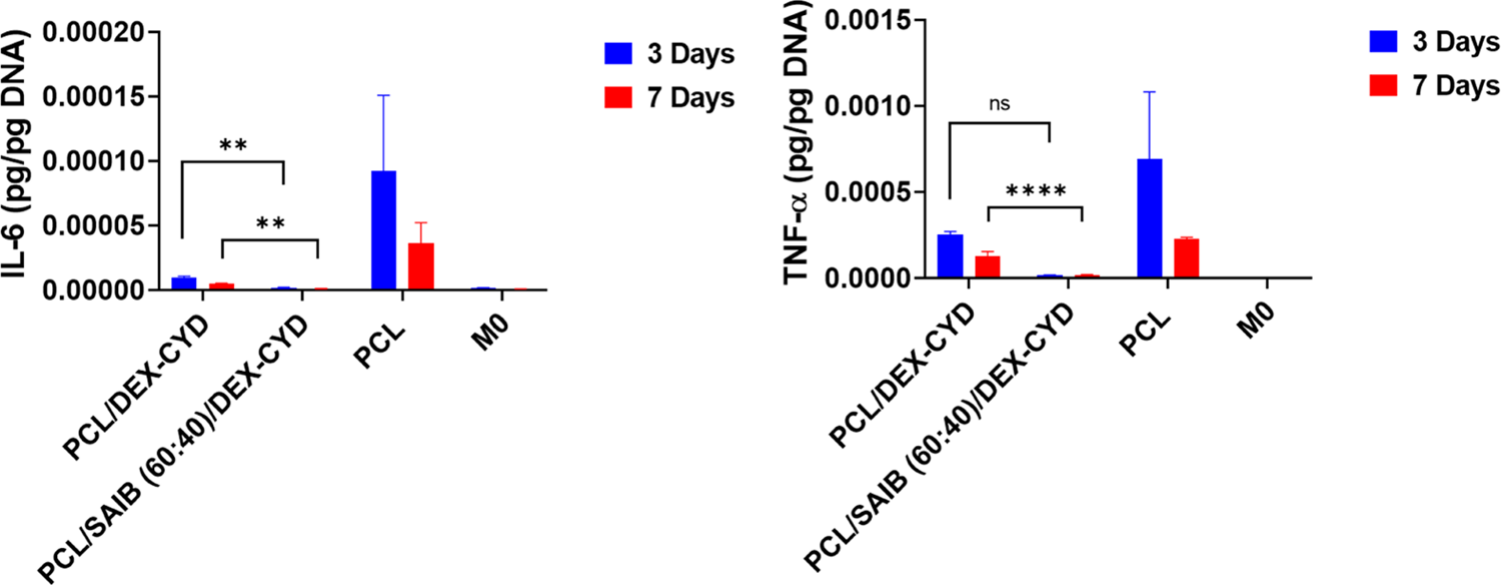
Quantification of pro-inflammatory cytokines in macrophage culture with the presence of 3D-printed PCL, PCL/DEX-CYD, and PCL/SAIB (60:40)/DEX-CYD scaffolds for 3 and 7 days. The concentrations of TNF-α and IL-6 were quantified by ELISA and normalized to cell DNA content. All data represent mean ± SD (*n* ≥ 3). ** *p* < 0.01, **** *p* < 0.0001.

The ability of the scaffold-released dexamethasone on the polarization of macrophages was subsequently investigated. Dexamethasone-loaded scaffolds were submerged into the M0 culture together with LPS and GM-CSF added into the media (except the M0 control). Culture with the dexamethasone-loaded scaffolds significantly reduced M1 cytokine IL-6 on days 3 and 7. TNF-α was also reduced in the dexamethasone-loaded scaffolds on day 3 and day 7 compared to their drug-free PCL counterparts (Figure 7). This suggested that the concentration of scaffold-released dexamethasone was in the effective concentration range. According to the release study, the estimated cumulative concentrations at days 3 and 7 are 44 and 56 nM, respectively, which falls within the effective range demonstrated in the bolus addition experiment.

### 3.6 Osteogenic Differentiation and Macrophage Polarization in Co-Cultures

**Figure 8:**
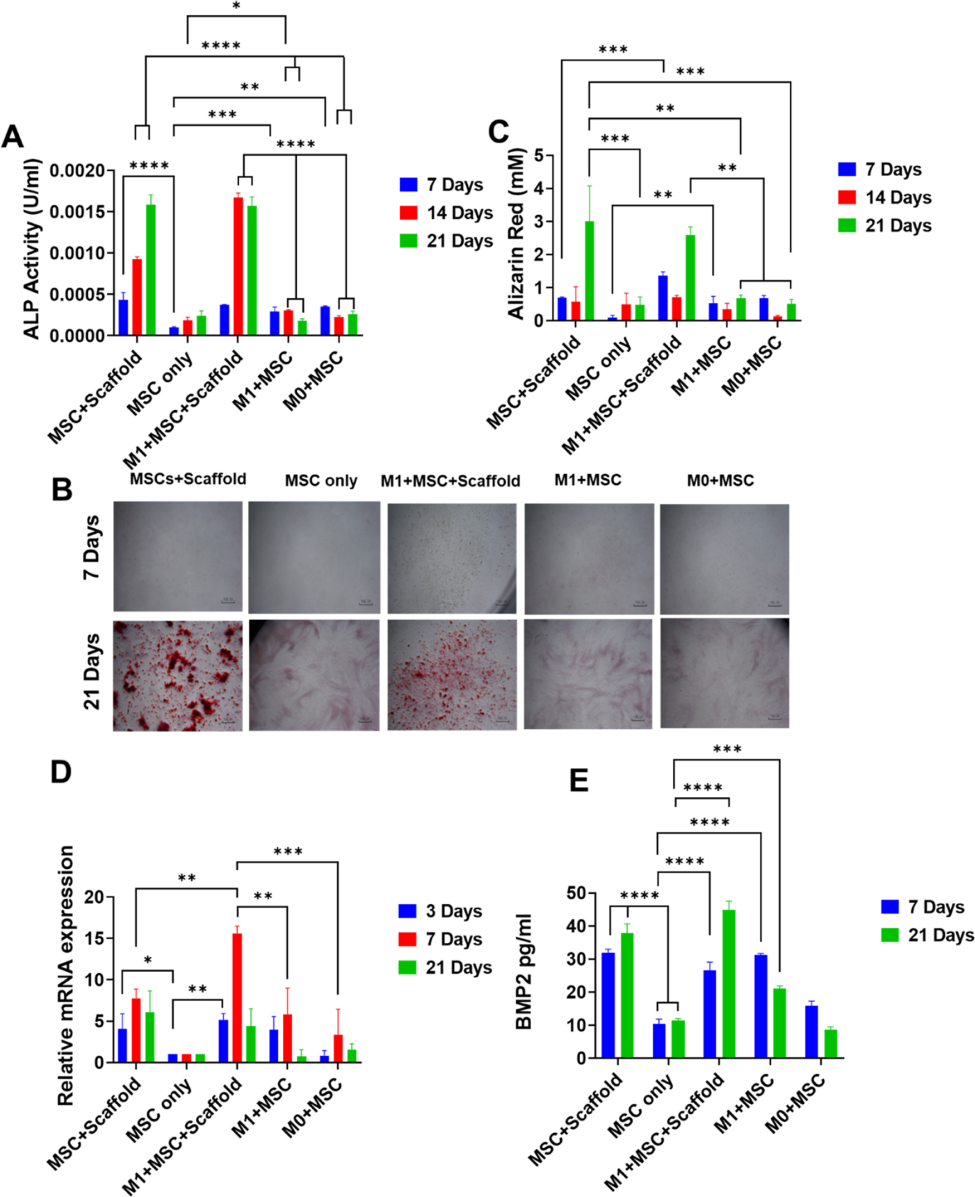
(A) ALP activity in different cultures over 7, 14, and 21 days (B) Alizarin red staining showing the mineralization in different cultures. Scale bar-100 μm. (C) Quantification of Alizarin red staining. (D) Gene expression of osteogenic marker RUNX2. (E) BMP-2 levels in different cultures. All data represent mean ± SD (*n* ≥ 3). * *p* < 0.05, ** *p* < 0.01, *** *p* < 0.001, **** *p* < 0.0001.

To investigate the interactions between macrophages and the osteogenic differentiation of MSCs, two cell types (5:1-macrophage/MSC) were co-cultured in the presence of 3D-printed scaffolds loaded with dexamethasone over 21 days. The cell ratio was chosen based on published data on the optimal ratio for maximizing osteogenic differentiation of MSCs.^14,17^ The osteogenesis of M1 + MSC co-culture in the presence of a 3D-printed scaffold was assessed by ALP activity, an early marker of osteogenic differentiation (Figure 8A). ALP was significantly increased in the cultures with the presence of dexamethasone-loaded scaffolds at day 7 compared to the MSC-only culture. This was likely due to the pro-osteogenic differentiation property of dexamethasone, which is typically added to cultures at 10–100 nM range for osteogenic differentiation via inducing Runx2 expression.^36^ Compared to MSC-only, the co-cultures with M1 or M0 also significantly enhanced the secretion of ALP at day 7. This might be due to the cytokines secreted by M1 and M0 cells that promoted osteogenic differentiation.^37,38^ There was no statistical difference in ALP secretion between the four cultures (excluding the MSC-only culture) on day 7.

ALP was higher in cultures with the presence of scaffolds at days 14 and 21, suggesting a stronger effect of dexamethasone on osteogenic differentiation compared to M1 macrophages. Between the two cultures in the presence of scaffolds, the inclusion of M1 cells significantly promoted ALP at day 14, indicating a synergistic effect of M1 and dexamethasone on osteogenic differentiation. However, ALP was at similar levels at day 21 for these two scaffold-included cultures, possibly due to the switching of the M1 to M2 phenotype caused by dexamethasone. M0 + MSC showed higher ALP than MSC-only culture at day 7, but no statistical difference at later time points. M1 + MSC showed higher ALP level at days 7 and 14 compared to MSC-only culture, but no difference at day 21. M1 + MSC and M0 + MSC showed similar levels (not statistically different) of ALP at all 3 time points.

Alizarin staining for measuring calcium content was used to determine the mineralization level of MSCs co-cultured with macrophages in the presence of dexamethasone-releasing scaffolds (Figure 8B,C). Matrix mineralization was significantly increased in cultures with dexamethasone-loaded scaffolds at day 21, which confirmed the osteogenic property of dexamethasone. At day 7, the mineralization was higher in the M1 + MSC co-culture compared to the MSC-only culture, followed by comparable mineralization between them on day 14 and day 21. This result correlated with the ALP data where the MSC + M1 co-culture showed a higher ALP level compared to the MSC-only culture at day 7. When the two cultures with scaffolds were compared, mineralization was higher at day 7 for the M1 + MSC + scaffold culture with no difference between them at day 14 and 21, suggesting an early effect of M1 on mineralization The M1 + MSC and M0 + MSC showed no difference at the 3 time points. These data demonstrated that the inclusion of M1 only promoted early-stage osteogenic differentiation. Late-stage osteogenic differentiation was dominated by the pro-osteogenic property of dexamethasone.

RUNX2 (a master osteogenic differentiation regulator) was higher for cultures with dexamethasone-releasing scaffolds and the M1 + MSC culture but not for the M0 + MSC, at day 3 compared to the MSC-only culture (Figure 8D). The M1 + MSC culture exhibited a similar RUNX2 level at day 3 compared to the two scaffold-containing cultures. The highest level of RUNX2 was the M1 + MSC + scaffold culture at day 7, which suggested a synergistic effect between M1 and dexamethasone on promoting osteogenic differentiation. This synergistic effect was also seen for ALP on a later time point (day 14) for the same culture. At day 21, the two scaffold-containing cultures showed similar levels of RUNX2.

BMP-2 has been reported as a soluble mediator for enhanced osteogenesis from macrophages.^39^ At day 7, M1 + MSC + scaffold, MSC + scaffold, M1 + MSC, and M0 + MSC showed a statistically higher level of BMP-2 compared to the MSC-only culture, suggesting both dexamethasone and macrophages increased the secretion of BMP-2 (Figure 8E). At day 21, M1 + MSC + scaffold, MSC + scaffold, and M1 + MSC showed significantly higher BMP-2 compared to the MSC-only culture, while M0 + MSC showed comparable BMP-2 level to MSC-only. At both days 7 and 21, M1 + MSC showed higher BMP-2 than M0 + MSC, demonstrating a stronger effect of M1 macrophages on the secretion of BMP-2 than M0 macrophages. In addition, the M1+MSC+scaffold culture showed higher BMP-2 compared to M1 + MSC at both days 7 and 21, and MSC + scaffold showed higher BMP-2 than M1 + MSC at day 21, demonstrating a stronger effect of dexamethasone on BMP-2-mediated enhanced osteogenic differentiation.

Taking the ALP, Alizarin red staining, RUNX2 expression, and BMP-2 data together, they showed a pro-osteogenesis effect of M1 macrophages at early time points. The osteogenic differentiation was dominated by dexamethasone at later time points. There was some synergistic effect in promoting osteogenic differentiation between M1 macrophages and dexamethasone at early/midtime points. The disappearance of the pro-osteogenic property by M1 may be due to the activation status change of the macrophages caused by dexamethasone.

**Figure 9:**
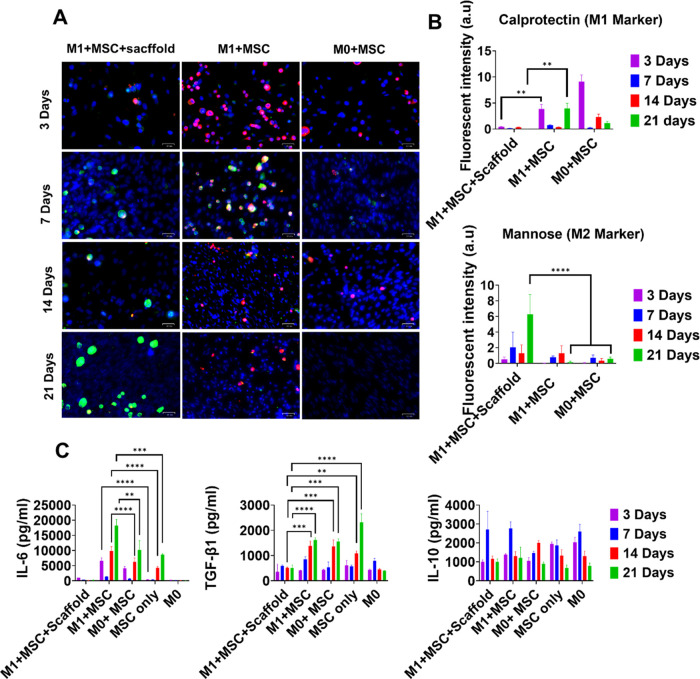
Macrophage activation status in co-cultures. (A) Immunostaining of M1 marker calprotectin (red) and M2 marker mannose receptor (green) and cell nuclei (blue). Scale bar, 100 μm. (B) Quantified fluorescence intensity of calprotectin and mannose receptors. (C) Quantifying pro-inflammatory (IL-6) and anti-inflammatory cytokines (TGF-β1 and IL-10). All data represent mean ± SD (*n* ≥ 3). * *p* < 0.05, ** *p* < 0.01, *** *p* < 0.001, **** *p* < 0.0001.

To verify this, we investigated the activation status of macrophages in the co-cultures over time. Our results showed the transition of macrophage activation status from M1 to M2 by the sustained release of dexamethasone (Figure 9). Surface markers for M1 (calprotectin) and M2 (mannose receptor) were fluorescently stained to characterize the different activation statuses of macrophages (Figure 9A). The M1 maker (calprotectin) was much lower at day 3 for the scaffold-containing media, which suggested a suppression of the M1 phenotype at early time points. At day 21, the expression of M1 surface marker was still lower in the scaffold-containing medium due to the sustained release of dexamethasone. On the other hand, the biggest difference in the M2 marker was at day 21, with the scaffold-containing media showing the highest mannose receptor level (Figure 9B). This suggested that the transition from M1 to M2 phenotype took a longer time compared to the relatively quick suppression of the M1 phenotype. MSCs also showed an immunosuppressive effect evidenced by lower calprotectin and higher mannose receptor at days 7 and 14 compared to day 3 for the M1 + MSC culture. However, this effect diminished at day 21, evidenced by the relative increase and decrease of calprotectin and mannose receptor, respectively.

In addition, two anti-inflammatory cytokines (IL-10, TGF-β1) and one pro-inflammation cytokine (IL-6) were measured by ELISA (Figure 9C). The scaffold culture suppressed IL-6 secretion at all time points. The M1 + MSC culture showed higher IL-6 at all 4 time points while M0 + MSC showed higher IL-6 only at days 3 and 7 compared to MSC-only, suggesting a simulated secretion of this cytokine from the MSCs by the macrophages. In addition, the M1 + MSC culture secreted more IL-6 compared to the M0 + MSC culture at all 4 time points, suggesting an effect of the macrophage phenotype on IL-6 secretion from MSCs. This elevated IL-6 level was correlated with the enhanced osteogenesis of MSCs by macrophages. In addition, the greater amount of IL-6 from M1 macrophages than M0 macrophages also correlated with the better osteogenic effect of M1 macrophages. This suggested that IL-6 is a possible factor that promoted osteogenesis. IL-6 is a pleiotropic cytokine. It is worth noting that OSM, which has been identified as an important soluble factor in macrophage-mediated enhanced osteogenesis, belongs to the IL-6 family cytokines as they all use the common signaling receptor subunit glycoprotein 130 kDa.^40^ On the other hand, it has previously shown that MSC-produced IL6 skews monocytes toward an anti-inflammatory phenotype.^41,42^

TGF-β1 was higher in the MSC-only, M1 + MSC, and M0 + MSC cultures compared to the scaffold culture at days 14 and 21 (Figure 9C). Dexamethasone at supraphysiological concentrations inhibits the proliferation (but not differentiation) of MSCs which also secrete TGF-β1.^35^ Therefore, the relatively lower level of TGF-β1 secretion in the scaffold culture was possibly attributed to this inhibitory effect. TGF-β1 was similar between the M1 + MSC and the M0 + MSC culture, suggesting a minimal effect of macrophage phenotype on the secretion of this cytokine. The MSC-only culture showed statistically higher TGF- β1 only at day 21 compared to M0 + MSC and M1 + MSC co-cultures. For IL-10, there was no difference among the five cultures at day 21.

**Figure 10:**
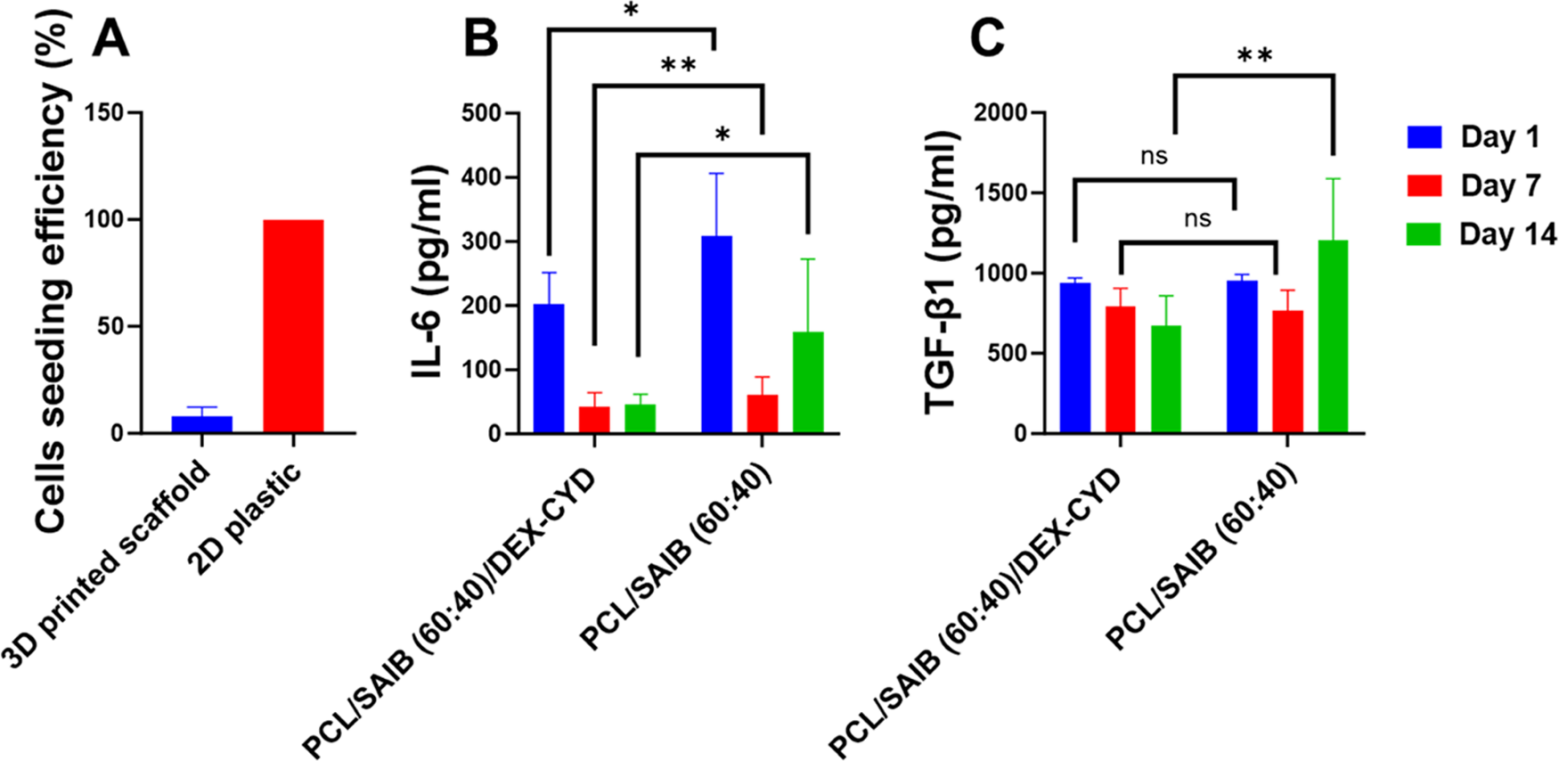
Responses of co-cultured macrophages and MSCs in 3D-printed scaffolds over 14 days. (A) Cell seeding efficiency in the porous scaffolds. (B) IL-6 and (C) TGF-β1 secretion from co-cultured cells at different times. All data represent mean ± SD (*n* ≥ 3). Comparison between drug-releasing scaffolds and drug-free scaffolds at each time point was made using Student’s *t* test. * *p* < 0.05, ** *p* < 0.01.

After testing the responses of cells cultured on 2D plastic wells with the presence of drug-releasing scaffolds, we then seeded and co-cultured macrophages and MSCs in scaffolds and characterized cell responses. The cell seeding efficiency was 8.2% compared to 2D plastic (100%) (Figure 10A). This is in agreement with what we discovered previously in which a large proportion of cells leaked through the porous scaffolds during cell seeding.^2^ Cell seeding efficiency was dependent on the pore size of the scaffolds.^2^ The remaining cells in the scaffolds were cultured for 14 days. The inflammatory cytokines and osteogenic markers were quantified by using ELISA. IL-6 was lower in the drug-releasing scaffolds compared to the drug-free scaffolds at all 3 time points, which agreed with the data on 2D plastic. TGF-β1 was lower in the drug-releasing scaffolds at day 14, which also agreed with the data for 2D plastic. Other soluble markers were mostly below the limit of quantification of the ELISA kits, which was probably due to the relatively low number of cells in the scaffolds (Figure S7). It is worth noting that seeding cells on scaffolds in vitro is likely different from how cells will contact scaffolds in vivo. We have recently demonstrated that the formation of cell contact with implanted scaffolds in vivo is different from in vitro experiments where cells are directly seeded on scaffolds.^42^ However, the characterization of cellular responses to these drug-releasing scaffolds in vivo is beyond the scope of this study.

## 4 Discussion

We reported for the first time the interactions between MSCs and macrophages in co-cultures with the sustained release of an anti-inflammatory and pro-osteogenic differentiation drug. We first achieved a sustained release of dexamethasone from PCL by adding SAIB to the polymer matrix, which only demonstrated a burst release in previous studies. We then characterized the osteogenic differentiation and macrophage polarization in different co-cultures.

We first identified SAIB as an effective excipient among the five compounds (Pluronic F127, Pluronic F68, Pluronic L31, and Span80) that were tested. SAIB has been used as a viscous injectable depot system for sustained drug release and elicited mild inflammation after intramuscular injection in rats.^43,44^ However, its effectiveness as an excipient in PCL to sustain drug release has not been tested before. These five excipients all have shown surfactant properties and exhibited a wide range of hydrophilicity/hydrophobicity. The relatively higher hydrophobicity of SAIB might be the reason that it showed the best ability in sustaining dexamethasone release. Hydration caused by the other excipients (Pluronic F127, Pluronic F68, Pluronic L31) was likely the main reason for the burst release of dexamethasone from the scaffolds.^45,46^ ToF-SIMS analysis demonstrated a more homogeneous distribution of dexamethasone with individual struts after adding SAIB to PCL. In contrast, dexamethasone was concentrated on the strut surface without SAIB. However, the exact molecular mechanism for this more homogeneous distribution will need further investigation. Sustained release of dexamethasone from poly(dimethylsiloxane) has been demonstrated.^47^ The release of zoledronic acid was demonstrated to be sustainable up to 30 days with cumulative release of approximately 50% from 3D-printed PCL/bioactive glass scaffolds.^48^ These studies suggest that the interaction between the polymer carrier and the drug plays an important role in the release profile.

Dexamethasone was selected as the releasing drug due to its dual properties: anti-inflammation and pro-osteogenic differentiation. The prolonged use of systemic corticosteroids such as dexamethasone is associated with potentially serious adverse effects. For example, prolonged use of dexamethasone has been shown to induce osteoporosis, possibly due to its ability to inhibit the proliferation of osteogenic precursors.^35^ However, this adverse effect was shown to be concentration-dependent, requiring >100 nM concentrations to inhibit the proliferation of osteogenic precursors.^35^ We hypothesized that a locally implanted scaffold can potentially reduce the systemic exposure of this drug while achieving nanomolar safe therapeutic concentrations at the desirable sites. The dexamethasone released from a 3D-printed PCL scaffold reduced proliferation but not the differentiation of human MSCs (Figure 5). The concentrations of released dexamethasone were slightly lower than the physiological level of cortisol (100–700 nM).^49^ However, these two glucocorticoids have different potencies with dexamethasone 17–30 times more potent than cortisol,^49^ meaning a supraphysiological level of released glucocorticoid from the scaffolds. This possibly explains the proliferation-inhibitory effect observed in our study and suggests a lower concentration of dexamethasone or less potent glucocorticoid to try in the future. The volume of media (10 mL) was fixed in both the release and the co-culture studies, hence affecting the drug concentration. During in vivo implantation, the local drug concentration will depend on the volume of the surrounding body fluids, how quickly they are replenished via diffusion and circulation, and how quickly the drug is eliminated in vivo. The final concentration of a drug in a scaffold may need to be tuned to be suitable for a specific implantation site. Another choice of anti-inflammatory drug is nonsteroidal anti-inflammatory drugs. However, their mechanism of action involves the COX-2/PGE2 process, which mediates the communication between macrophages and MSCs. Therefore, NSAIDs that interrupt this communication will compromise bone healing, which has been reported in the literature.^50,51^

Various studies have confirmed the importance of macrophages on osteogenesis. Depleting macrophages using liposome clodronate or in genetically modified mice lacking macrophages led to compromised bone formation.^52,53^ However, the exact macrophage phenotype that stimulates osteogenesis has some conflicting data. Some studies reported only M1 promoted osteogenic differentiation.^15^ Other reports have shown all three phenotypes (M0, M1, and M2) showed enhanced osteogenic differentiation of MSCs.^14,15,54,55^ Our data showed a pro-osteogenic effect of M1 macrophages at early time points. This agrees with previous in vitro and in vivo reports showing that macrophages contributed to osteogenesis early during the inflammatory phase of fracture healing. A combination of factors is likely responsible for macrophage-mediated enhanced osteogenesis. OSM, BMP-2, and PGE2 have been the most recognized soluble mediators for macrophage-mediated enhanced osteogenesis.^56^ Our data showed that both dexamethasone and M1 increased the level of BMP-2 compared to the MSC-only culture. This confirms the role of BMP-2 in enhanced osteogenesis. At the longer time point (21 days), the effect of M1 on BMP-2 secretion diminished, possibly due to the immunosuppressive effect of MSCs and dexamethasone on macrophages. The cellular source of BMP-2 was not used in our study. Both MSC and macrophages have been reported to be able to secret BMP-2.^57^ Future efforts will involve an investigation of the source and timing of BMP-2.

M0 macrophages were also able to promote ALP and matrix mineralization at day 7, but this effect diminished at later time points. This partially agrees with previous studies.^14,58^ Our data also suggested a better osteogenic effect by M1 macrophages compared with their M0 counterparts (evidenced by RUNX2 and BMP-2 data). The later stage of osteogenesis (mineralization) of MSCs was the highest in the two scaffold cultures, suggesting that the promineralization effect was dominated by dexamethasone. The released dexamethasone switched the phenotype of macrophages from M1 to M2 over time, evidenced by the increased level of the mannose receptor. As our experiment did not include an M2 + MSC culture, the comparison between M2 macrophages and dexamethasone in their effect on enhancing mineralization was not obtained. The effect of the dexamethasone-release scaffold on osteogenic differentiation may come from the combined effect of the drug and the M2 macrophages induced by it. As the initial inflammation and its resolution are both important for successful bone healing, future efforts will be needed to better control the timing of the released drug. For example, the released drug should not inhibit the initial beneficial inflammatory response but rather prevent chronic inflammation. Moreover, applying this concept will need careful consideration as surgery and implantation of the scaffold could happen over a range of timings after the formation of bone defects, which may require an initial assessment of the inflammatory status at the time of surgery.

It is also well known that MSCs possess immunosuppressive properties via paracrine signaling or cell–cell contact.^59^ For example, MSCs have been shown to switch activated M1 macrophages to an M2 phenotype via prostaglandin E2.^60,61^ MSC-borne chemokines, CCL2 in particular, have been found to modulate the phenotype of macrophages.^62^ Factors secreted by pro-inflammatory and anti-inflammatory macrophages activated the immunomodulatory potential of MSCs.^63,64^ Inflammation-primed hMSCs exhibited higher immunomodulatory capacity compared to nonprimed cells.^63,64^ All microphage phenotypes (M0,M1,M2) were demonstrated to be potent chemotactic stimulators for MSCs.^65^ Immuno-regulatory effects of placental MSCs have been found to be mediated by soluble molecules, such as IL10 and TGF-β1, acting partially via glucocorticoid receptor and progesterone receptor.^66^ In our study, the immunosuppressive effect of MSCs on M1 macrophages was transitory and to a lesser degree compared to dexamethasone, which showed a dramatic effect on switching macrophage phenotype from M1 to M2 (evidenced by calprotectin and mannose receptor). The M1 + MSC and M0+MSC co-cultures showed a higher level of IL-6 compared to the MSC-only culture, suggesting the enhanced secretion of IL-6 by macrophages (Figure 9C). IL-6 is a pleiotropic cytokine and is one of the cytokines associated with M1 macrophages. MSC can also secrete IL-6 when they were primed by TLR4.^67^ Interestingly, IL-6 has been found to enhance the polarization of M2 macrophages but required the involvement of IL-4 and IL-13.^68^ IL-6 was also found to promote osteogenic differentiation in bone marrow-MSCs via an autocrine/paracrine IL-6/IL-6R/STAT3 signaling pathway,^69^ and to promote migration of osteogenic stromal cells.^70^ The increase in IL-6 secretion in macrophage(M0/M1) + MSC co-cultures suggested the role of this cytokine in macrophage-mediated enhanced osteogenesis. The dexamethasone scaffold completely blocked the secretion of IL-6 while still promoting osteogenesis, suggesting an overriding effect via this glucocorticoid. Future effort will be needed to elucidate how the secretion of IL-6 from MSCs is induced by macrophages and how dexamethasone supressed it.

## 5 Conclusions

For the first time, the interaction between MSCs and macrophages in co-cultures with controlled release of an anti-inflammatory and pro-osteogenic differentiation drug was investigated. We successfully produced 3D-printed scaffolds that released dexamethasone in the nanometer range in a more sustained manner, which was not previously possible. The dual-property dexamethasone both promoted osteogenic differentiation and suppressed pro-inflammatory M1 macrophages in MSC-macrophage co-cultures. We found that M1 macrophages contributed to osteogenesis at early time points, and this effect diminished at later time points. M1 macrophages showed a stronger osteogenic effect than M0 macrophages. The late-stage mineralization was dominated by dexamethasone. Dexamethasone promoted more secretion of BMP-2 compared to macrophages, while M1 macrophages had more BMP-2 secretion than M0 macrophages. IL-6 secretion from MSCs was enhanced by macrophages, particularly M1 macrophages, suggesting the pro-osteogenic property of this cytokine in macrophage-induced osteogenesis. Our findings highlighted the critical role of the inflammatory response in the osteogenic differentiation of osteoprogenitor cells and the importance of studying the interactions between inflammatory and bone-forming cells. In addition, our work highlights the potential of the controlled release of drugs from implantable scaffolds to modulate both osteogenic differentiation and macrophage activation status in bone fracture healing.

## Supporting Information Available

Differential scanning calorimetry thermograms of different ink formulations (Figure S1); parameters determined by DSC for different ink formulations (Table S1); cumulative and actual release of dexamethasone from 3D-printed scaffolds of PCL/ F127 (70:30)/DEX-CYD, PCL/F127 (60:40)/DEX-CYD, PCL/ F68 (70:30)/DEX-CYD, and PCL/F68 (60:40)/DEX-CYD (Figure S2); cumulative and actual release of dexamethasone from 3D-printed scaffolds of PCL/ DEX-CYD, PCL/L31 (70:30)/ DEX-CYD, and PCL/L31/ (60:40)/DEX-CYD (Figure S3); cumulative and actual release of dexamethasone from 3D-printed PCL/ DEX-CYD, PCL/Span80 (70:30)/ DEX-CYD, and PCL/Span80 (60:40)/ DEX-CYD (Figure S4); polarization of M0, M1 and M2 macrophages derived from THP-1 monocytes and M0, M1, and M2 cytokine expression in RPMI single medium (Figure S5); polarization of M0, M1, and M2 macrophages derived from THP-1 monocytes and M0, M1, and M2 cytokine expression in a mixed-media RPMI/α-MEM (Figure S6); quantification of cytokines from the macrophage-MSC coculturing in scaffolds (Figure S7); one-way ANOVA comparison for Figure 5 in main publication (Tables S2 and S3); one-way ANOVA comparison for Figure 6 in main publication (Tables S4–S6); one-way ANOVA comparison for Figure 7 in main publication (Tables S7 and S8); one-way ANOVA comparison for Figure 8 in main publication (Tables S9–S12); and one-way ANOVA comparison for Figure 9 in main publication (Tables S13–S15) (PDF)
