# Supplementary material for: Sustained Release of Dexamethasone from 3D-Printed Scaffolds Modulates Macrophage Activation and Enhances Osteogenic Differentiation
Source: ACS Appl Mater Interfaces. 2023 Nov 28;15(49):56623–38. doi: 10.1021/acsami.3c09774 (PMC10726309; doi:10.1021/acsami.3c09774)
Supplement: Supplementary file 1 — am3c09774_si_001.pdf [file am3c09774_si_001.pdf]

Supporting information

Sustained release of dexamethasone from 3D printed scaffolds modulates macrophage activation and enhances osteogenic differentiation

Majed Majrashi[1,4], Anna Kotowska[1], David Scurr[1], Jacqueline M. Hicks[3], Amir Ghaemmaghami[2*], Jing Yang[1,4*]

1. School of Pharmacy, University of Nottingham, UK
2. School of Life Sciences, University of Nottingham, UK
3. Nanoscale and Microscale Research Centre, University of Nottingham, Nottingham, UK
4. Biodiscovery Institute, University of Nottingham, UK

* Corresponding authors: Jing Yang (jing.yang@nottingham.ac.uk); Amir Ghaemmaghami (amir.ghaemmaghami@nottingham.ac.uk)

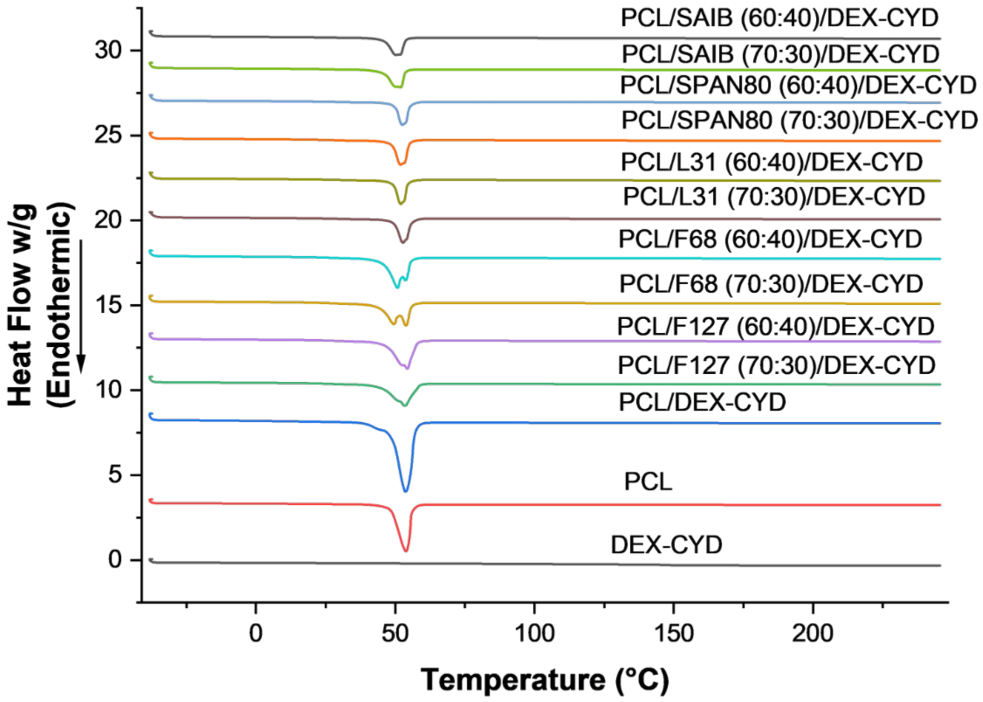

Figure S1: Differential scanning calorimetry thermograms of different ink formulations.

Table S1: Parameters determined by DSC for different ink formulations.

Sample	T_m (°C)	ΔH_m	X_c (%)
PCL	57.53	65.450	46.92
PCL/DEX-CYD	56.08	84.36	60.47
PCL/F127 (70:30)/DEX-CYD	53.57	59.21	42.44
PCL/F127 (60:40)/DEX-CYD	54.17	67.56	48.43
PCL/F68 (70:30)/DEX-CYD	54.06	58.89	42.22
PCL/F68 (60:40)/DEX-CYD	52.38	69.29	49.67
PCL/L31 (70:30)/DEX-CYD	52.67	39.78	28.52
PCL/L31 (60:40)/DEX-CYD	51.87	36.35	26.06
PCL/Span80 (70:30)/DEX-CYD	51.12	39.96	28.85
PCL/Span80 (60:40)/DEX-CYD	50.04	37.46	26.85
PCL/SAIB (70:30)/DEX-CYD	51.98	39.17	28.08
PCL/SAIB (60:40)/DEX-CYD	49.71	36.19	25.94

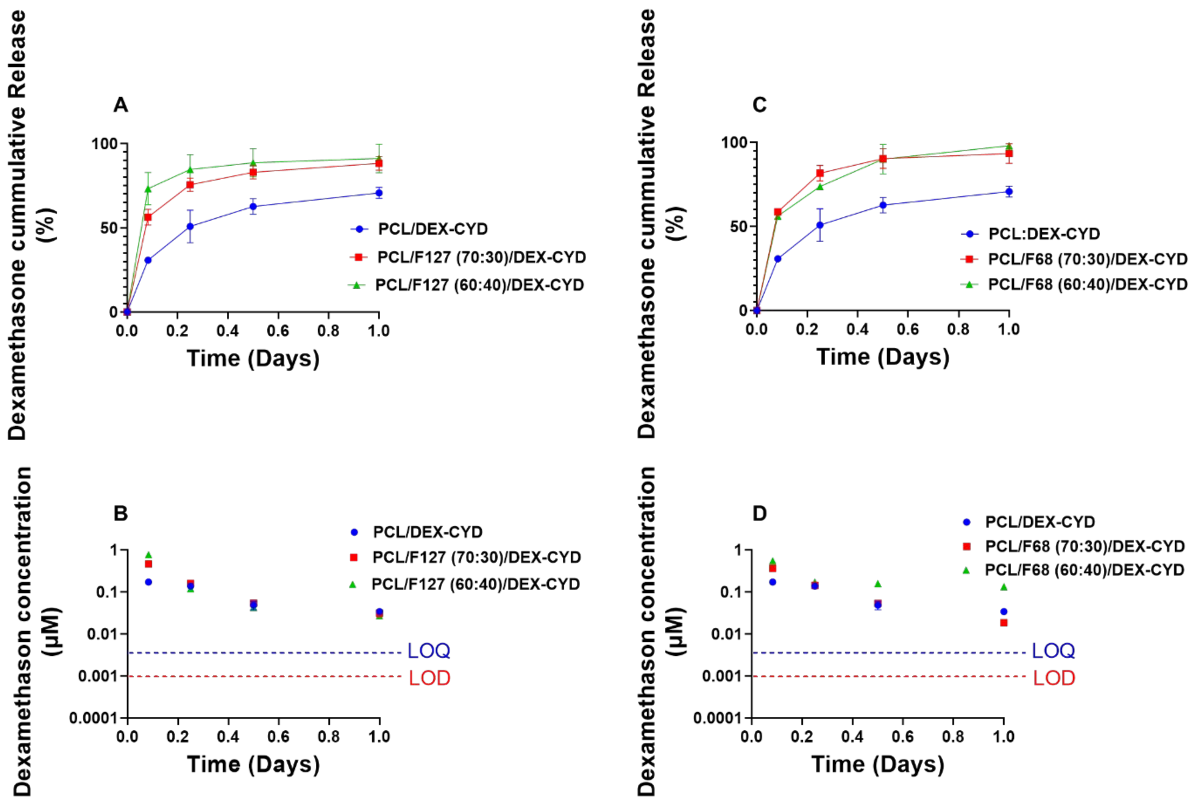

Figure S2: Cumulative and individual release at each collection time point from 3D printed scaffolds in 10mL PBS. (**A**) Cumulative release from 3D printed scaffolds of PCL/ F127 (70:30)/DEX-CYD and PCL/F127 (60:40)/DEX-CYD. (**B**) Individual release at each collection time point. (**C**) Cumulative release from PCL/ F68 (70:30)/DEX-CYD, and PCL/F68 (60:40)/DEX-CYD. (**D**) Individual release at each collection time point. All data represent mean ± SD (n=3).

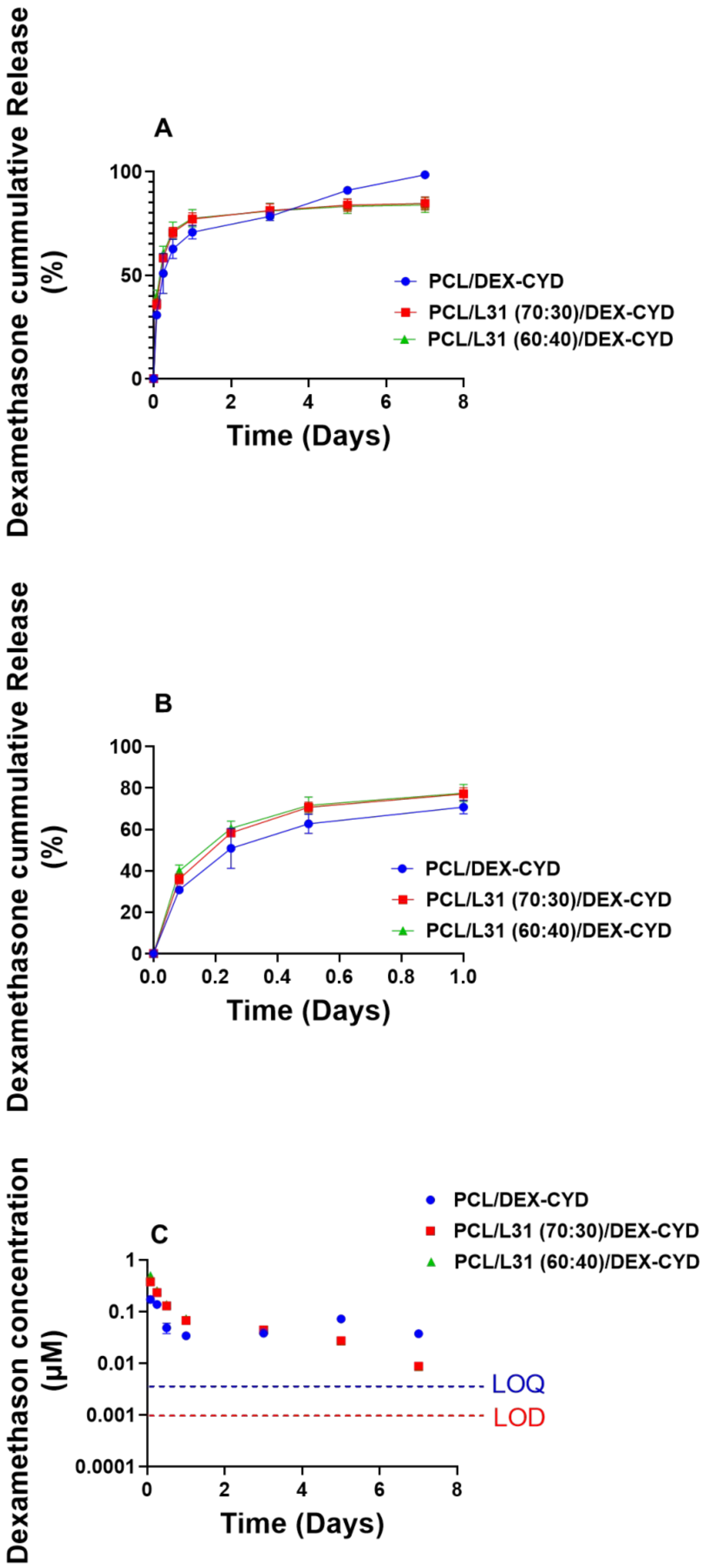

Figure S3: Cumulative and individual release at each collection time point from 3D printed scaffolds in 10mL PBS. (**A**) Cumulative release from 3D printed scaffolds of PCL, PCL/L31 (70:30), and PCL/L31 (60:40) loaded with 0.17% wt/wt of DEX-CYD. (**B**) Cumulative release over 1 day. (**C**) Individual release at each collection time point. Release medium was collected every two days after day 1. All data represent mean ± SD (n=3).

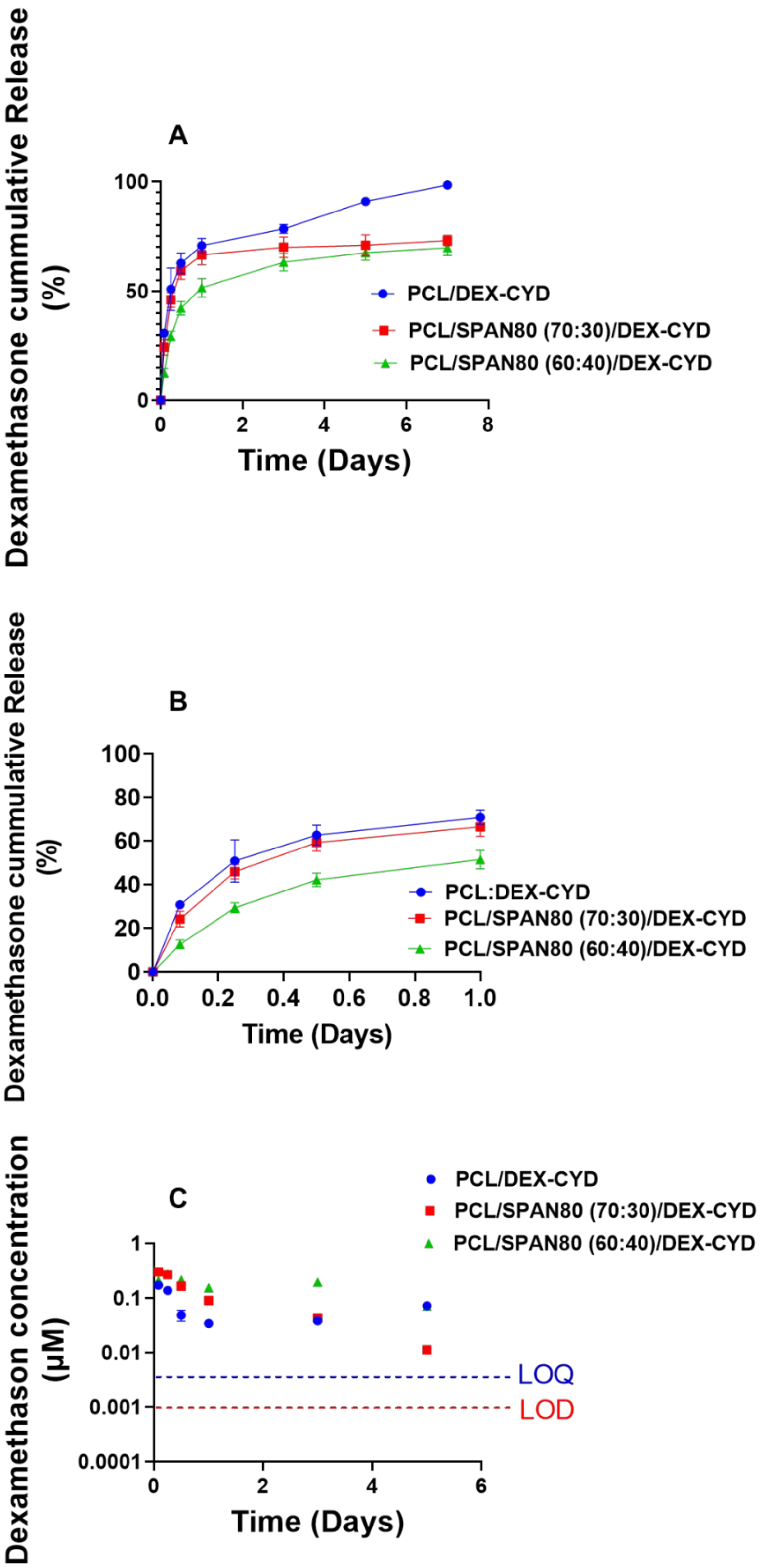

Figure S4: Cumulative and individual release at each collection time point from 3D printed scaffolds in 10mL PBS. (**A**) Cumulative release percentage of 3D printed scaffolds of PCL, PCL/Span80 (70:30), and PCL/Span80 (60:40) loaded with 0.17% wt/wt of DEX-CYD. (**B**) Cumulative release percentage over 1 day. (**C**) Individual release at each collection time point. Release medium was collected every two days after day 1. All data represent mean ± SD (n=3).

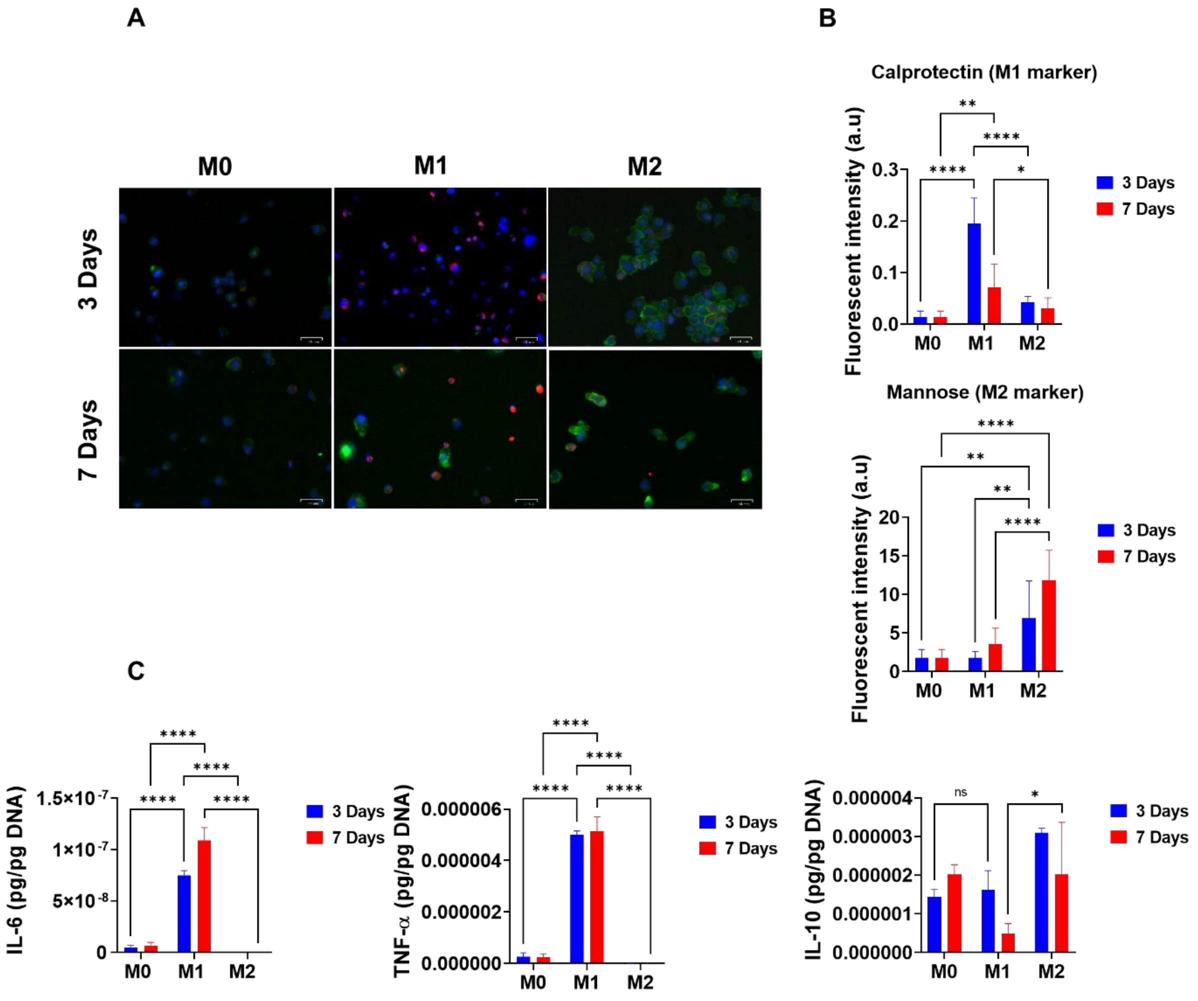

Figure S5: The polarisation of M0, M1 and M2 macrophages derived from THP-1 monocytes and M0, M1, and M2 cytokine expression. (**A**) Immunostaining of macrophages polarised at days 3 and 7 in RPMI single-media. The scale bar is 40 µm, respectively. (**B**) Fluorescence intensity was quantified by measuring the integrated density of Calprotectin (M1) and Mannose (M2) positive cells; this was normalized against the total number of cells (DAPI) by ImageJ. (**C**) pro-inflammatory cytokines TNF-α, IL-6, and anti-inflammatory IL-10 quantification of macrophages polarised in RPMI single media were quantified by ELISA and normalized to DNA content. All data represent mean ± SD (n=3). Statistical analysis was performed by one-way analysis of variance with Tukey's post hoc test indicated statistical differences with * $p < 0.05$, ** $p < 0.01$, *** $p < 0.001$, and **** $p < 0.0001$, respectively.

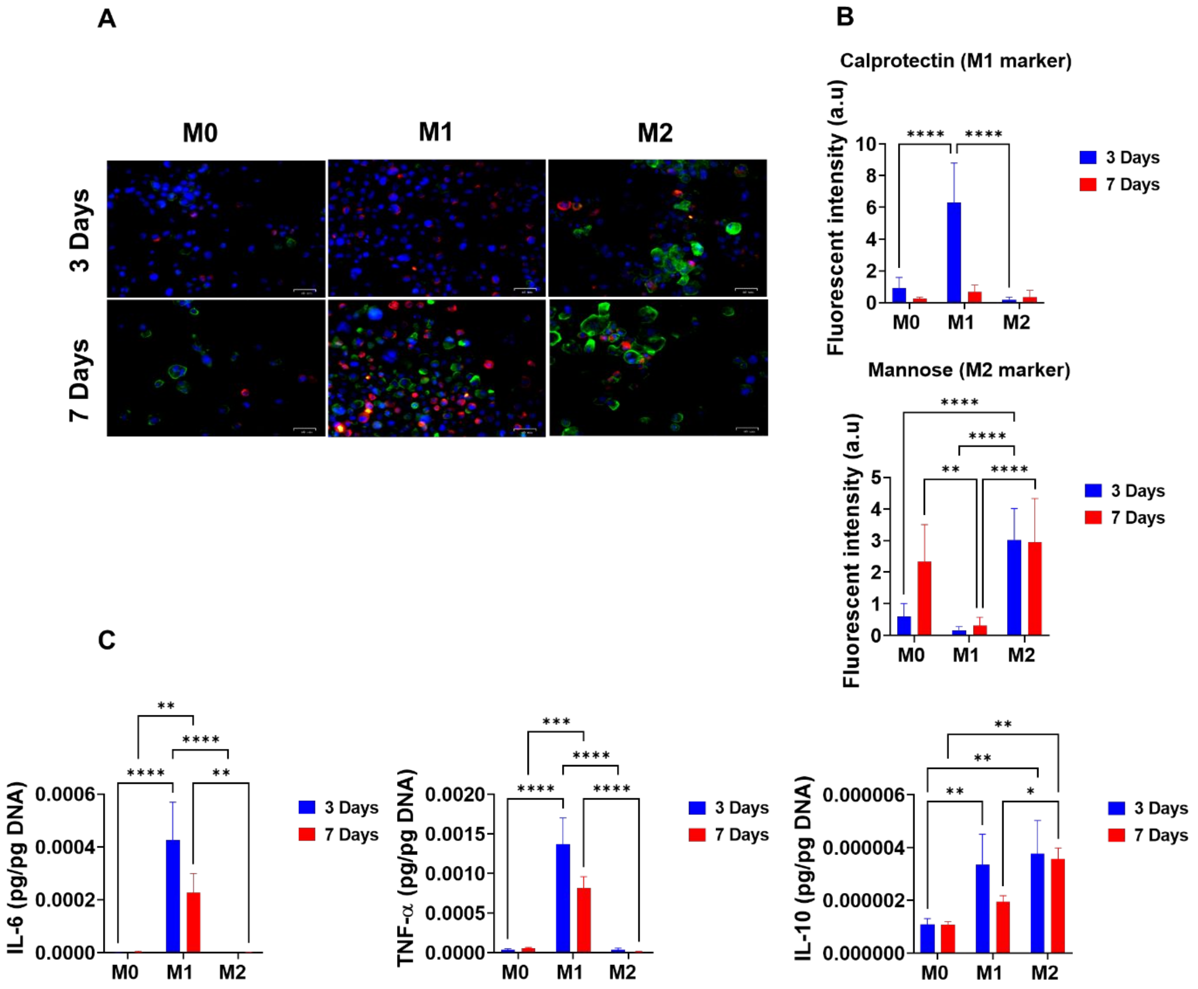

Figure S6: The polarisation of M0, M1 and M2 macrophages derived from THP-1 monocytes and M0, M1, and M2 cytokine expression. (**A**) Immunostaining of macrophages polarised at days 3 and 7 in a mixed-media (RPMI:α-MEM). The scale bar is 40 µm, respectively. (**B**) Fluorescence intensity was quantified by measuring the integrated density of Calprotectin (M1) and Mannose (M2) positive cells; this was normalized against the total number of cells (DAPI) by ImageJ. (**C**) Quantification of pro-inflammatory cytokines TNF-α, IL-6, and anti-inflammatory IL-10 from polarised macrophages in a mixed-medium (RPMI:α-MEM). All data represent mean ± SD (n=3). Statistical analysis was performed by one-way ANOVA with Tukey's post hoc. * $p < 0.05$, ** $p < 0.01$, *** $p < 0.001$, and **** $p < 0.0001$

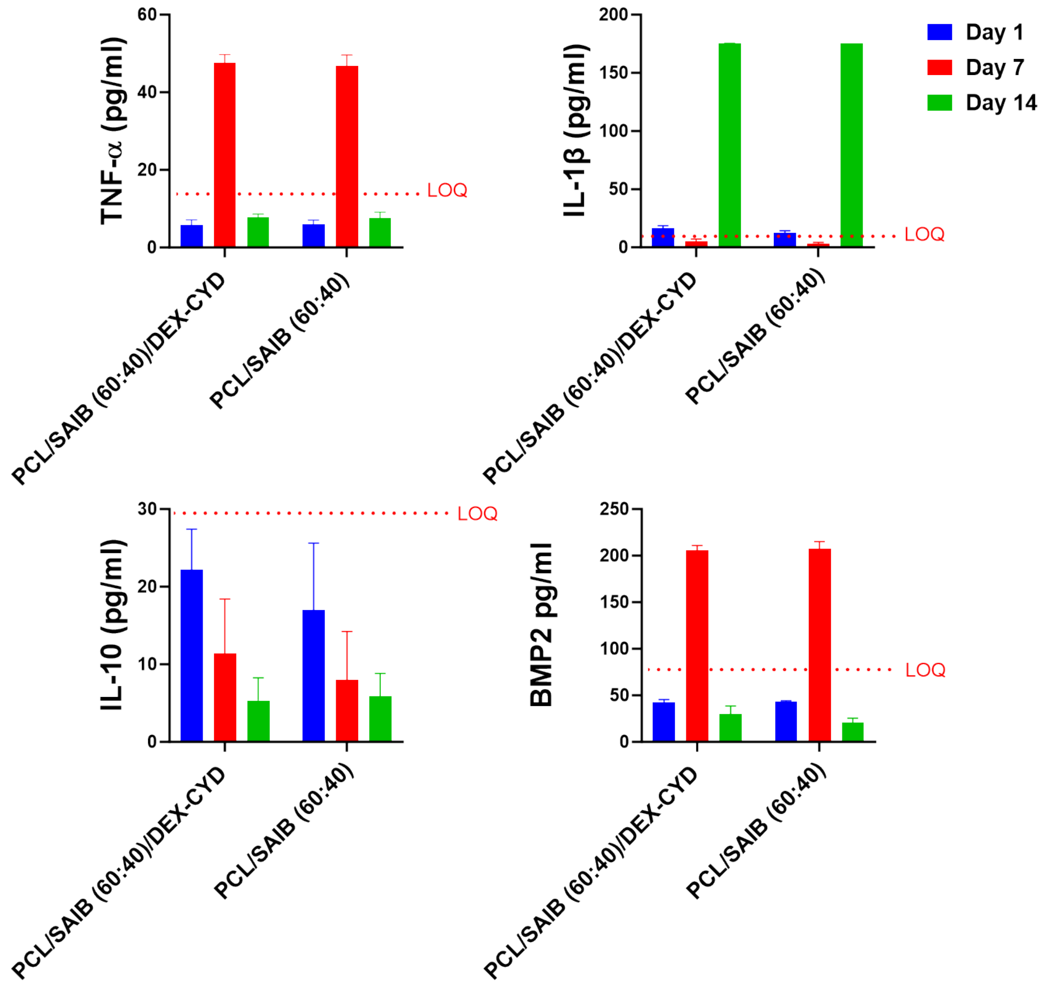

Figure S7. Quantification of cytokines from the macrophage-MSC co-culturing in scaffolds. Most values were below Limit of quantification (LOQ).

Table S2: (Figure 5 in the main text) One-way ANOVA Pairwise comparison MSC DNA in Co Culture media (RPMI:α-MEM 50:50) with or without of PCL/SAIB (60:40)/DEX-CYD scaffold at days 7,14, and 21.

MSC + Scaffold	Summary
7 Days vs. 14 Days	*
7 Days vs. 21 Days	***
14 Days vs. 21 Days	*
**MSC Only**	**Summary**
7 Days vs. 14 Days	ns
7 Days vs. 21 Days	ns
14 Days vs. 21 Days	ns

Table S3: (Figure 5 in the main text) One-way ANOVA Pairwise comparison of MSC DNA in RPMI with or without of PCL/SAIB (60:40)/DEX-CYD scaffold at days 7, 14, and 21.

MSC + Scaffold	Summary
7 Days vs. 14 Days	ns
7 Days vs. 21 Days	**
14 Days vs. 21 Days	**
**MSC Only**	**Summary**
7 Days vs. 14 Days	***
7 Days vs. 21 Days	****
14 Days vs. 21 Days	*

Table S4: (Figure 6 in the main text) One-way ANOVA Pairwise comparison of the effect of dexamethasone concentration on IL1β production from macrophage. THP-1 cells were first differentiated into M0 macrophages. LPS, GM-CSF and dexamethasone were added to the media (Except the M0 control) at days 3 and 7.

Samples	3 Days	7 Days
100 μM vs. 10μM	ns	ns
100 μM vs. 1 μM	ns	ns
100 μM vs. 0.1 μM	ns	ns
100 μM vs. 0.01 μM	ns	****
100 μM vs. 0.μM	ns	****
100 μM vs. M 0	****	**
10μM vs. 1 μM	ns	ns
10μM vs. 0.1 μM	ns	ns
10μM vs. 0.01 μM	ns	****
10μM vs. 0.μM	ns	****
10μM vs. M 0	****	*
1 μM vs. 0.1 μM	ns	ns
1 μM vs. 0.01 μM	ns	****
1 μM vs. 0.μM	ns	****
1 μM vs. M 0	****	ns
0.1 μM vs. 0.01 μM	ns	****
0.1 μM vs. 0.μM	ns	****
0.1 μM vs. M 0	****	**
0.01 μM vs. 0.μM	ns	****
0.01 μM vs. M 0	****	****
0.μM vs. M 0	****	****

Table S5: (Figure 6 in the main text) One-way ANOVA Pairwise comparison of the effect of dexamethasone concentration on IL-6 production from macrophage. THP-1 cells were first differentiated into M0 macrophages. LPS, GM-CSF and dexamethasone were added to the media (Except the M0 control) at days 3 and 7.

Samples	3 Days	7 Days
100 µM vs. 10µM	ns	ns
100 µM vs. 1 µM	ns	ns
100 µM vs. 0.1 µM	ns	ns
100 µM vs. 0.01 µM	*	*
100 µM vs. 0.µM	****	****
100 µM vs. M 0	***	ns
10µM vs. 1 µM	ns	ns
10µM vs. 0.1 µM	ns	ns
10µM vs. 0.01 µM	**	*
10µM vs. 0.µM	****	****
10µM vs. M 0	**	ns
1 µM vs. 0.1 µM	ns	ns
1 µM vs. 0.01 µM	**	**
1 µM vs. 0.µM	****	****
1 µM vs. M 0	**	ns
0.1 µM vs. 0.01 µM	ns	*
0.1 µM vs. 0.µM	****	****
0.1 µM vs. M 0	****	ns
0.01 µM vs. 0.µM	****	****
0.01 µM vs. M 0	****	***
0.µM vs. M 0	****	****

Table S6: (Figure 6 in the main text) One-way ANOVA Pairwise comparison of the effect of dexamethasone concentration on TNF-α production from macrophage. THP-1 cells were first differentiated into M0 macrophages. LPS, GM-CSF and dexamethasone were added to the media (Except the M0 control) at days 3 and 7.

Samples	3 Days	7 Days
100 µM vs. 10µM	ns	ns
100 µM vs. 1 µM	ns	*
100 µM vs. 0.1 µM	ns	*
100 µM vs. 0.01 µM	ns	ns
100 µM vs. 0.µM	**	ns
100 µM vs. M 0	ns	****
10µM vs. 1 µM	ns	ns
10µM vs. 0.1 µM	ns	ns
10µM vs. 0.01 µM	ns	ns
10µM vs. 0.µM	**	ns
10µM vs. M 0	ns	***
1 µM vs. 0.1 µM	ns	ns

1 µM vs. 0.01 µM	ns	ns
1 µM vs. 0.µM	**	***
1 µM vs. M 0	ns	ns
0.1 µM vs. 0.01 µM	ns	ns
0.1 µM vs. 0.µM	**	***
0.1 µM vs. M 0	ns	*
0.01 µM vs. 0.µM	ns	**
0.01 µM vs. M 0	*	**
0.µM vs. M 0	***	****

Table S7: (Figure 7 in the main text) One-way ANOVA Pairwise comparison of the effect of dexamethasone concentration on IL-6 production from macrophage. THP-1 cells were first differentiated into M0 macrophages. LPS, GM-CSF and PCL/DEX-CYD or PCL/SAIB (60:40)/DEX-CYD were added to the media (Except the M0 control) at days 3 and 7.

Samples	3 Days	7 Days
PCL/DEX-CYD vs. PCL/SAIB (60:40)/DEX-CYD	**	**
PCL/DEX-CYD vs. PCL	ns	ns
PCL/DEX-CYD vs. M0	*	****
PCL/SAIB (60:40)/DEX-CYD vs. PCL	ns	ns
PCL/SAIB (60:40)/DEX-CYD vs. M0	ns	ns
PCL vs. M0	ns	ns

Table S8: (Figure 7 in the main text) One-way ANOVA Pairwise comparison of the effect of dexamethasone concentration on TNF-α production from macrophage. THP-1 cells were first differentiated into M0 macrophages. LPS, GM-CSF and PCL/DEX-CYD or PCL/SAIB (60:40)/DEX-CYD were added to the media (Except the M0 control) at days 3 and 7.

samples	3 Days	7 Days
PCL/DEX-CYD vs. PCL/SAIB (60:40)/DEX-CYD	ns	****
PCL/DEX-CYD vs. PCL	ns	***
PCL/DEX-CYD vs. M0	*	****
PCL/SAIB (60:40)/DEX-CYD vs. PCL	ns	****
PCL/SAIB (60:40)/DEX-CYD vs. M0	**	ns
PCL vs. M0	ns	****

Table S9: **(**For figure 8 in main text): One-way ANOVA Pairwise comparison for ALP production in MSCs co-cultured with M1 macrophages in the presence of 3D printed PCL/SAIB (60:40)/DEX-CYD scaffolds over 7, 14, and 21 days.

Samples	7 Days	14 Days	21 Days
MSC + Scaffold vs. MSC only	****	****	****

MSC + Scaffold vs. M1+MSC + Scaffold	ns	****	ns
MSC + Scaffold vs. M1+MSC	*	****	****
MSC + Scaffold vs. M0+MSC	ns	****	****
MSC only vs. M1+MSC + Scaffold	***	****	****
MSC only vs. M1+MSC	**	*	ns
MSC only vs. M0+MSC	***	ns	ns
M1+MSC + Scaffold vs. M1+MSC	ns	****	****
M1+MSC + Scaffold vs. M0+MSC	ns	****	****
M1+MSC vs. M0+MSC	ns	ns	ns

Table S10: (For figure 8 in main text): One-way ANOVA Pairwise comparison for Alizarin Red production in MSCs co-cultured with M1 macrophages in the presence of 3D printed PCL/SAIB (60:40)/DEX-CYD scaffolds over 7, 14, and 21 days.

Samples	7 Days	14 Days	21 Days
MSC + Scaffold vs. MSC only	**	ns	***
MSC + Scaffold vs. M1+MSC + Scaffold	***	ns	ns
MSC + Scaffold vs. M1+MSC	ns	ns	**
MSC + Scaffold vs. M0+MSC	ns	ns	***
MSC only vs. M1+MSC + Scaffold	****	ns	**
MSC only vs. M1+MSC	*	ns	ns
MSC only vs. M0+MSC	**	ns	ns
M1+MSC + Scaffold vs. M1+MSC	***	ns	**
M1+MSC + Scaffold vs. M0+MSC	**	ns	**
M1+MSC vs. M0+MSC	ns	ns	ns

Table S11: (For figure 8 in main text): One-way ANOVA Pairwise comparison for Bone morphogenic protein-2 (BMP-2) production in MSCs co-cultured with M1 macrophages in the presence of 3D printed PCL/SAIB (60:40)/DEX-CYD scaffolds over 7, 14, and 21 days.

Samples	7 Days	21 Days
MSC + Scaffold vs. MSC only	****	****
MSC + Scaffold vs. M1+MSC + Scaffold	*	**
MSC + Scaffold vs. M1+MSC	ns	****
MSC + Scaffold vs. M0+MSC	*	****
MSC only vs. M1+MSC + Scaffold	****	****
MSC only vs. M1+MSC	****	***
MSC only vs. M0+MSC	**	ns
M1+MSC + Scaffold vs. M1+MSC	*	****
M1+MSC + Scaffold vs. M0+MSC	****	****
M1+MSC vs. M0+MSC	****	****

Table S12 : (For figure 8 in main text): One-way ANOVA Pairwise comparison for RUNX2 gene expression in MSCs co-cultured with M1 macrophages in the presence of 3D printed PCL/SAIB (60:40)/DEX-CYD scaffolds over 3, 7, and 21 days.

Samples	3 Days	7 Days	21 Days
MSC only vs. MSC+ Scaffold	*	**	*
MSC only vs. M1+MSC + Scaffold	**	****	ns
MSC only vs. M1+MSC	*	ns	ns
MSC only vs. M0+MSC	ns	ns	ns
MSC+ Scaffold vs. M1+MSC + Scaffold	ns	**	ns
MSC+ Scaffold vs. M1+MSC	ns	ns	*
MSC+ Scaffold vs. M0+MSC	*	ns	ns
M1+MSC + Scaffold vs. M1+MSC	ns	**	ns
M1+MSC + Scaffold vs. M0+MSC	**	***	ns
M1+MSC vs. M0+MSC	*	ns	ns

Table S13: (For figure 9 in main text) One-way ANOVA Pairwise comparison for IL-6 production in MSCs co-cultured with M1 macrophages in the presence of 3D printed PCL/SAIB (60:40)/DEX-CYD scaffolds over 3, 7, 14, and 21 days.

Samples	3 Days	7Days	14 Days	21 Days
M1+MSC+Scaffold vs. M1+MSC	****	****	****	****
M1+MSC+Scaffold vs. M0+ MSC	****	**	****	***
M1+MSC+Scaffold vs. MSC only	ns	ns	***	***
M1+MSC+Scaffold vs. M0	ns	ns	ns	ns
M1+MSC vs. M0+ MSC	***	****	**	***
M1+MSC vs. MSC only	****	****	****	***
M1+MSC vs. M0	****	****	****	****
M0+ MSC vs. MSC only	****	**	ns	ns
M0+ MSC vs. M0	****	***	****	***
MSC only vs. M0	ns	ns	***	***

Table S14: (For figure 9 in main text) One-way ANOVA Pairwise comparison for TGF-β1 production in MSCs co-cultured with M1 macrophages in the presence of 3D printed PCL/SAIB (60:40)/DEX-CYD scaffolds over 3, 7, 14, and 21 days.

Samples	3 Days	7 Days	14 Days	21 Days
M1+MSC+Scaffold vs. M1+MSC	ns	ns	***	****
M1+MSC+Scaffold vs. M0+ MSC	ns	ns	***	***
M1+MSC+Scaffold vs. MSC only	ns	ns	**	****
M1+MSC+Scaffold vs. M0	ns	ns	ns	ns
M1+MSC vs. M0+ MSC	ns	ns	ns	ns
M1+MSC vs. MSC only	ns	ns	ns	**
M1+MSC vs. M0	ns	ns	***	****
M0+ MSC vs. MSC only	ns	ns	ns	**
M0+ MSC vs. M0	ns	ns	***	****
MSC only vs. M0	ns	ns	**	****

Table S15: (For figure 9 in main text) One-way ANOVA Pairwise comparison for IL-10 production in MSCs co-cultured with M1 macrophages in the presence of 3D printed PCL/SAIB (60:40)/DEX-CYD scaffolds over 3, 7, 14, and 21 days.

Samples	3 Days	7 Days	14 Days	21 Days
M1+MSC+Scaffold vs. M1+MSC	ns	ns	ns	ns
M1+MSC+Scaffold vs. M0+ MSC	ns	ns	**	ns
M1+MSC+Scaffold vs. MSC only	***	ns	ns	ns
M1+MSC+Scaffold vs. M0	***	ns	ns	ns
M1+MSC vs. M0+ MSC	ns	ns	*	ns
M1+MSC vs. MSC only	*	ns	ns	ns
M1+MSC vs. M0	**	ns	ns	ns
M0+ MSC vs. MSC only	***	ns	*	ns
M0+ MSC vs. M0	***	ns	*	ns
MSC only vs. M0	ns	ns	ns	ns
